# Ubiquity of Aviation Ultrafine Particles and Lubrication Oil Compounds Near Zurich Airport

**DOI:** 10.1021/acs.est.5c18458

**Published:** 2026-04-23

**Authors:** Sarah Tinorua, Benjamin. T. Brem, Zachary C. J. Decker, Jay G. Slowik, Peter A. Alpert, Markus Ammann, André S. H. Prévôt, Michael Bauer, Suneeti Mishra, Michael Götsch, Joerg Sintermann, Martin Gysel-Beer

**Affiliations:** † PSI Center for Energy and Environmental Sciences, Villigen 5232, Switzerland; ‡ Canton of Zurich, AWEL - Amt für Abfall, Wasser, Energie und Luft, Zurich 8090, Switzerland

**Keywords:** ultrafine particles (UFPs), airport emissions, aircraft turbine engine lubrication oil, tricresyl phosphate (TCP), extractive electrospray ionization (EESI) mass spectrometry

## Abstract

Ultrafine particles (UFPs) are a major air quality concern because their small diameter (<100 nm) allows them to reach the lungs’ alveolar regions, causing adverse health effects. Civil aviation and airports are important sources of UFPs in urban areas. In this study, a one-month intensive measurement campaign in November 2022, 1 km downwind of Zurich Airport, revealed that high UFP number concentrations up to 300 000 cm^–3^ originate solely from aircraft operations. These emissions are either advected downwind of the airport or mixed downward during aircraft landings overhead. The measurements confirm that most aviation-related UFPs are volatile with geometric mean diameters <20 nm. Under certain conditions, they can rapidly grow up to ∼40 nm, while their volatile particle number fraction drops from ∼0.9 to ∼0.1. Online mass spectrometry shows that engine lubrication oil signals closely track aviation-related high UFP levels, enabling attribution of high UFP concentration events to aviation emissions. Multiyear measurements at the site further show that airport emissions dominate daytime UFP concentrations for ∼30% of the time across all wind directions. The widespread presence of UFPs and related organophosphate oil compounds poses a health concern for communities near airports that regulators should address.

## Graphical abstract

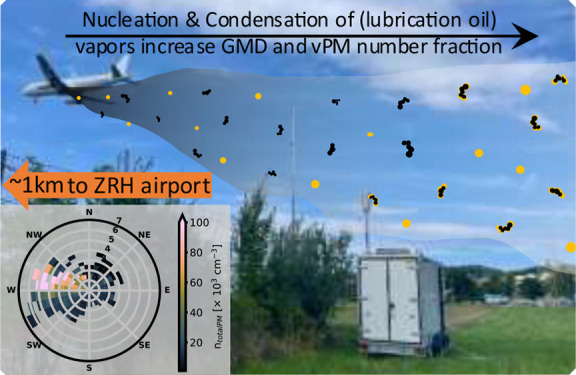

## Introduction

Ultrafine particles (UFPs), defined as particles smaller than 100 nm in diameter, can reach the circulatory system of the human body, where they can cause respiratory, cardiovascular, and even neurological issues.^1,2^ Airport emissions were shown to contribute significantly to elevated UFP number concentrations in urban areas, such as Copenhagen, Los Angeles, and Warwick.^3–5^ However, among the pollutants emitted by a civil aviation turbofan engine, only the emissions of the major gaseous pollutants (i.e., carbon monoxide (CO), nitrogen oxides (NO_*x*_), hydrocarbons, and recently, nonvolatile particulate matter (nvPM) mass and number) are regulated globally by the International Civil Aviation Organization.^6^ Furthermore, the 2023 revision of European air quality legislation recommends UFP monitoring in terms of number concentration near pollution hotspots, such as airports, traffic, and harbors.^7^ UFPs from aircraft exhaust plumes are a mix of nvPM, i.e., black carbon particles formed by incomplete combustion, and volatile PM (vPM).^8^ Their size and emission factors depend on engine thrust, environmental conditions, fuel composition, and engine type.^9,10^ While nvPM is immediately formed within the engine’s combustor, resulting in fractal-like BC aggregates,^11^ vPM forms from emitted vapors, which become supersaturated as the exhaust cools during dilution. This phase transition can occur either by condensation onto existing nvPM particles or by nucleation followed by condensational growth to form fresh vPM particles. The nucleation pathway typically leads to a dominant nucleation mode (diameter <20 nm) in the UFP particle number size distribution.

In near-field aviation plumes (within a few hundred meters before dispersion), vPM is primarily composed of sulfates formed through gas phase oxidation of fuel sulfur and to a lesser extent of an organic fraction made of unburned fuel and, in some cases, lubrication oil (lube oil) compounds.^8,12^ Lube oil has been previously identified as an important mass fraction in aircraft engine exhaust using a high-resolution time-of-flight aerosol mass spectrometer (HR-ToF AMS).^13–15^ However, the major part of UFPs emitted by aircraft engines is in the nucleation mode, especially at low thrusts,^16^ which is not accessible to AMS measurements. Oil compounds were found to dominate the composition of UFPs < 30 nm in the near field at the runway, they could be quantified even 5 km away from Frankfurt Airport, and they were shown to contribute to nucleation processes.^17,18^ Both studies applied offline mass spectrometry on impactor-collected samples with a time resolution of 18–57 h, which hinders the assessment of diurnal patterns and the coemission of lube oil with other pollutants.

Although quantitative understanding of the physicochemical properties of aircraft-emitted UFPs at the engine exit plane and in the near field has increased, their far-field dynamics and characteristics remain less well characterized. The main exception is the well-established presence of high concentrations of nucleation mode particles. In particular, the interaction between airport operational aspects and plume dynamics with ambient conditions, including meteorological factors and background pollution, which govern UFP properties in the far field, requires further investigation.

This work overcomes previous limitations by providing online in situ field measurements of volatile and nonvolatile UFP number size distributions. It further reveals the presence of several unique aviation emission tracers (e.g., engine lubrication oil compounds), simultaneously detected at a monitoring site located next to a schoolhouse, 1 km downwind of Zurich International Airport. The influence of airport operational patterns on UFP properties disentangles the contributions from aviation, road traffic, and other sources and finally shines light on plume aging processes and lubrication oil compounds.

## Materials and Methods

### Measurement Site

Our aerosol instrumentation, described later, was set up in an open field in the city of Kloten from October 25 to December 3, 2022. The location is next to the air quality monitoring station Kloten Feld, operated by OSTLUFT, the intercantonal authority for air quality monitoring in eastern Switzerland,^19^ and within 50 m of a primary school. The site is qualified as a suburban background station, meaning that road traffic would not directly influence the measured parameters.^20^ However, the site is surrounded by several potential UFP sources, including the major A-4 highway at a distance of 400 m and busy roads located 300–500 m away, the Kloten industrial area approximately 700 m away, and the focus of this study, Zurich International Airport, 1 km away. With around 31.2 million passengers in 2024, Zurich Airport was the 18th busiest airport in the world.^21^ In 2022, when these measurements took place, Zurich Airport hosted 216’585 aircraft movements,^22^ a lower number compared to 2024 (261′103 aircraft movements), which is attributed to the slow recovery from the 2020 COVID pandemic. The airport has three runways: two in the north-west to south-east axis (numbers 16/34 and 14/32) and one crossing the runway 16/34 on the west-to-east axis (10/28), as can be seen in Figure S1. The east end of runway 28 of Zurich Airport, on which flights generally take off from 06:00 to 21:00 on weekdays (from 06:00 to 20:00 on weekends, respectively), is located approximately 1 km west of our measurement site. In the evening after 21:00 on weekdays (20:00 on weekends, respectively), most landings are also facilitated on this runway until the stop of aircraft movements at 23:00, due to noise regulations. The prevailing westerly (247.5–292.5°) wind directions (27% of measurement time), which commonly occur during the airport’s operating hours (see Figure S1b), allow the sampling of emissions associated with air traffic at Zurich Airport in the well-mixed dispersion regime of the engine plumes. In addition, downmixed plumes are also detected from aircraft passing directly over the site when landing on runway 28. Other local influences are expected to be stronger when the wind blows from the south–southeast (112.5–202.5°), bringing road traffic, industrial emissions, and a lower impact of airport-related emissions. Southeast (112.5–157.5°) winds may carry less emissions from industry and traffic but more from the nearby residential areas, such as domestic wood burning and cooking. Road traffic on the motorway and the major roads surrounding the measurement site is monitored at several locations, as shown in Figure S1a. Hourly number counts are resolved by vehicle category (heavy trucks, light trucks, buses, and normal cars) and are provided for both directions of each monitoring point. In this study, the road traffic data are defined as the sum of all vehicles passing by the monitoring points and going in both directions.

### Air Inlets and Sampling Manifolds

A container and a mobile trailer hosted the instrumentation at the site. The container is part of the regulatory OSTLUFT network operated by the Canton of Zurich (AWEL) and is permanently installed at the site, while the trailer was deployed solely for the measurement campaign. Each facility had separate PM_1_ and gas phase inlets, 4.1–4.3 m above ground. The PM_1_ inlet and flow distribution in the measurement container were compliant with EN 16976:2024 for the particle number measurement of ambient aerosol (see the OSTLUFT report for details on the sample inlets^19^). A standardized PM_1_ sampling head (Digitel HVS-PM1) in combination with a custom-made flow distribution and transmission system specifically designed for low PM losses was utilized in the trailer. The gas phase measurements followed Swiss national guidelines^23^ and used a coated glass manifold with additional bypass flow and PTFE tubing for sample transmission. The inlet of the extractive electrospray ionization long-time-of-flight mass spectrometer (EESI-LToF-MS) was connected to the aerosol inlet via carbon-impregnated conductive Teflon tubing.

### Total and Nonvolatile UFP Measurements

The UFP size distribution was measured by a scanning mobility particle sizer (SMPS, Model 3938, TSI Inc.), composed of a nanodifferential mass analyzer (nanoDMA, Model 3085A, TSI Inc.), coupled with a water-based condensation particle counter (CPC, Model 3789, TSI Inc.), measuring particles between 4.96 and 101.8 nm in diameter. A standalone CPC Model 3789 (TSI Inc.) was used in parallel to measure the total PN concentration between 2.2 and 1000 nm. These instruments were continuously operated as part of the air quality monitoring program by AWEL. Three additional butanol-based CPCs, with lower cutoff diameters of 1, 2.5, and 7 nm, respectively (Models 3750–50, 3756, and 3750-CEN) were placed in our trailer to complete the PN concentration measurements. A comparison of the total number concentration data reported by the two CPCs, shown in Figure S2, reveals that the CPC model 3756 operated in our trailer behind the customized inlet measured lower concentrations than the CPC model 3789, operated in the AWEL container. Data were filtered out when the CPCs' upper count limit was reached (200,000 and 300,000 cm^–3^ for CPC 3756 and CPC 3789, respectively). The relative difference between the CPCs increased as the modal diameter of the UFP size distribution decreased, reflecting stronger diffusion losses. These losses were accounted for only in the container’s CPC 3789 data, and systematic uncertainties from differing CPC sampling conditions (e.g., working fluids, upstream sampling trains, and inlets) can further explain the differences between the two CPCs. Given the still overall good level of agreement with an average ratio of 0.78 between the two instruments, we did not apply any further corrections to the trailer’s CPC 3756 data.

The UFP size distributions reported include corrections for SMPS internal losses (CPC counting efficiency, diffusion and neutralizer losses), but also for diffusion losses within the sampling train from the inlet to the instrument hook-up.^24^ An example of the size dependent correction factor applied can be found in Figure S3.

From November 16 to November 29, the trailer hosted the nvPM size distribution measurements that used a catalytic stripper CS (CS015, Catalytic Instruments) placed in front of a standard SMPS (SMPS3938, TSI Inc.) that consisted of a long DMA (Model 3081, TSI Inc.) in combination with a butanol-based ultrafine CPC (Model 3776, TSI Inc.). The catalytic stripper removes semivolatile inorganic and organic compounds from the total PM by heating the sample flow up to 350 °C followed by catalytic oxidation and transformation of the vaporized material into the gas phase.^25^ In terms of losses, the nvPM size distribution data were additionally corrected with an empirical particle loss correction function provided by the catalytic stripper instrument application note.^26^ The nvPM number fraction is defined as the number concentration ratio of nvPM to total PM, both measured across the UFP diameter range between 6.4 and 102 nm.

The geometric mean diameter (GMD) was determined by fitting the UFP size distribution with a log-normal function over its whole measured size range. In addition to the campaign data set, the AWEL UFPs size distribution data over a two year period from 2022 and 2023 were further processed for a robust characterization of diurnal patterns segregated by wind sector and comparisons between weekdays and weekends, where the campaign data could not have provided good enough statistics.

### Oil Tracer Measurements

The PM chemical composition was measured with an EESI-LToF-MS, which provides real-time near-molecular-level measurements of particulate organic matter, including the UFP size range^27^: the molecular formula can be retrieved without information on its molecular structure or isomeric forms. The EESI-LToF-MS provides data with a 1 s time resolution, which is high enough to allow data segregation by wind sectors. The version used in this study has been developed at PSI^28^ and includes a conventional electrospray plume to intersect and extract sampled aerosol particles in a low-temperature, soft ionization scheme. In this study, a mixture of 50:50 water/acetonitrile doped with 100 ppm of sodium iodide (NaI) was used as the electrospray solution. Acetonitrile aids the extraction of the nonpolar/hydrophobic organic PM fraction. The molecules reported here were all detected as Na^+^ adducts. More details about this EESI-LToF-MS can be found in previous literature.^29–31^

Data were pre-averaged onto 30 s intervals and peak-fitted using Tofware version 3.2.3 (Tofwerk AG, Thun, Switzerland). Particles were continuously sampled through an extruded carbon denuder, which removes organic gases, thereby decreasing the instrument background and avoiding erroneous interpretation of sticky gases as a particle-phase signal. A high efficiency particulate air (HEPA) filter with a switching valve allowed frequent measurements of the instrument background. A cycle of 3 min of direct sampling followed by 3 min of background measurement through the filter was continuously performed. A continuous baseline signal was obtained by linear interpolation between the averages of each background measurement. Next, the EESI-LToF-MS aerosol sample raw data points were averaged over the 3 min intervals, and the corresponding PM composition signals were obtained by subtracting the interpolated baseline signals. The PM composition signals were mostly near the lower limit of detection. The sensitivity of the EESI-LToF-MS at the molecular level is highly dependent on the particle size and the chemical structure, but also on the instrumental setup (spray nozzle position and physics).^29–31^ Assessing the instrument’s sensitivity in an online setting is not practically feasible. Therefore, the PM composition signals were classified in terms of signal to noise, specifically signal as a multiple of the standard deviation of the background (σ_bck_; separately for each compound). A compound data point above 3 σ_bck_ would reflect a higher signal detected by the EESI-LToF-MS than that if it was below 2 σ_bck_. Signals below 1 σ_bck_ are not discernible from random background noise.

The lube oil markers analyzed here included pentaerythritol (PA) and dipentaerythritol (DPA) esters with carbon chain lengths between C_25_ and C_60_, and tricresyl phosphate (TCP, formula C_21_H_21_O_4_P), which have a prevalence in Mobile Jet Oil II (ExxonMobil, Irving, TX, USA), the most common jet engine lubrication oil in the global market.^32^ In addition, trimethylolpropane (TMP) esters found in the jet oils from BP/Eastman were further included in the analysis. Table S1 presents the lube oil markers analyzed in this study with their raw formula, the mass-to-charge (*m*/*z*) ratio of the Na^+^ adduct, and the name used herein. Decker et al.^33^ reported a poor signal-to-noise ratio for esters in Mobil Jet oil II with high carbon number, leading to nonmeasurable EESI-LToF-MS signals for esters > C_36_. For the same reason, the esters selected for this study are C_27_ or smaller. In addition, other compounds usually used as ambient tracers but not related to aircraft engine emissions, including nicotine or alphapinene oxidation products, were further included in the analysis. The EESI-LToF-MS provides molecular composition and quantitative relative concentration changes over time. However, absolute quantification of analyte concentrations is typically impractical in complex matrices due to considerable variation in compound-specific sensitivities (e.g., a factor of 5 variation even among isomers), with particle size effects on absolute sensitivity in the UFP range presenting a further complication in the present analysis.^31,30^ In this study, EESI-LToF-MS signals are used to identify jet engine lubricating oil tracers and assess the variability in those tracer signals in comparison to those of other commonly detected ambient aerosol tracers.

### (Trace) Gas measurements

Regulatory measurements of nitric oxide (NO) and nitrogen dioxide (NO_2_) (with a chemiluminescence detector), sulfur dioxide (SO_2_) (via UV absorption), and carbon dioxide (CO_2_) (using nondispersive infrared, i.e., NDIR) were part of the continuous observations by OSTLUFT.^34^ In addition, a MIRO multigas analyzer (MGA-10 GP, MIRO Analytical AG) was operated during the intense period of this study. The MGA-10 GP is based on mid-infrared laser absorption spectroscopy using quantum cascade lasers as light sources and allows the selective and sensitive quantification of ten gases: carbon monoxide (CO), CO_2_, methane (CH_4_), NO, NO_2_, ozone (O_3_), nitrous oxide (N_2_O), SO_2_, ammonia (NH_3_), and water vapor (H_2_O).^35^

## Results and Discussion

### Enhanced UFP Concentrations at the Kloten Site Are Driven by Aviation Emissions

UFPs observed at the Kloten site may originate from many different sources, e.g., new particle formation, road traffic, domestic heating, aviation, or transported from distant sources. To investigate source contribution, we first assess the wind sector dependence of UFP properties measured at this site. Figure 1 shows polarplots of the averaged UFP number concentrations, *n*_totalPM_, and the averaged nucleation mode fraction, defined as the ratio of the UFP number concentrations in the nucleation mode, *n*_totalPM,nucl_, to *n*_totalPM_, segregated by daytime, evening, and nighttime.

**Figure 1:**
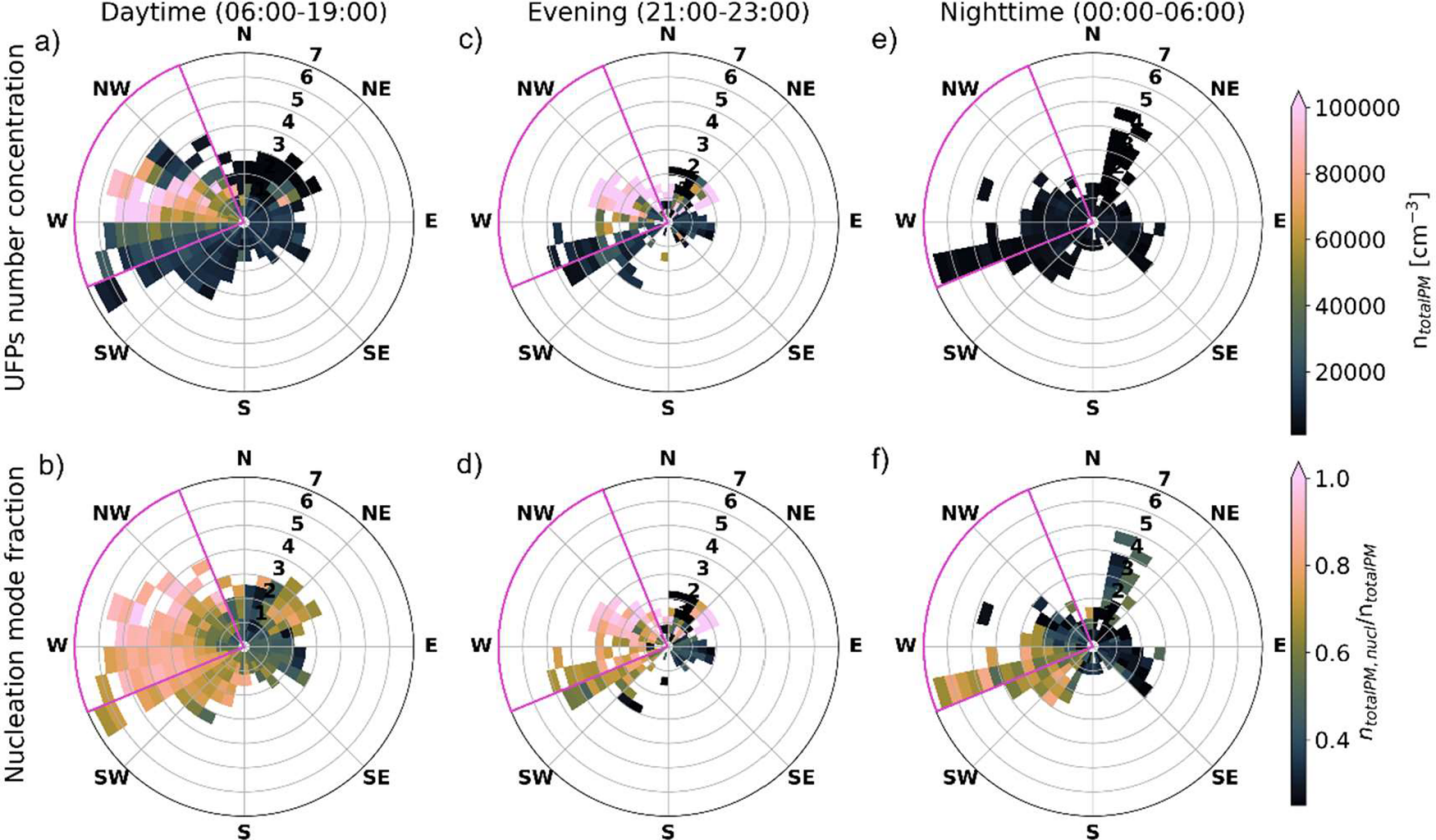
Polarplots of UFP number concentrations for diameters between 4.96 and 101.2 nm on the top panels and nucleation mode fraction on the bottom panel for (a,b) daytime (from 06:00 to 19:00 LT), (c,d) evening (from 21:00 to 23:00 LT), and (e,f) nighttime (from 00:00 to 06:00 LT). The measurements covered the period between October 25 and December 3, 2022. The radial axis represents the wind speed, and the downwind airport sector is colored purple (247.5–337.5°).

During the day (from 06:00 to 19:00) and the evening (21:00 to 23:00), the highest UFP concentrations, up to 300,000 cm^–3^ (10 min average value), were measured when the site was downwind of the airport (W-NNW, i.e., 247.5 to 337.5°, marked with a purple frame on Figure 1), under medium to strong wind speeds (Figure 1a). The vast majority of these UFPs are smaller than 20 nm in diameter, indicated by the high nucleation mode fraction of around 0.8–0.9 in the downwind sector (Figure 1b). As a reminder, during daytime, aircraft generally take off from east to west on runway 28, while from 21:00 to 23:00, this runway is used by the aircraft to land, thus flying over the measurement site. After 23:00, no more aircraft movements occur, except for very few delayed arrivals, due to the nighttime shutdown of the airport.

In the evening, notably, even higher UFP number concentrations than in the downwind sector were observed in the NNW to ENE wind sector (326.25–78.75°, Figure 1c). In the evening time window, most landings occur on runway 28 (Figure S1), which happens with aircraft passing directly over the measurement site during approach with a vertical distance of as little as ∼80 m. Therefore, these high UFP number concentrations are attributed to downmixing of emissions from aircraft passing over the site during approach to the airport, as further discussed later. The nucleation mode fraction of the UFPs downmixed from landing overpass was >0.9 (Figure 1d), so even higher than for the UFP plume in the downwind sector (Figure 1b).

Interestingly, the WSW-SW (236.25–202.5°) wind sector also featured an enhanced nucleation mode fraction during the nighttime, outside the scheduled flight operation (Figure 1f). Likely, emergency helicopters and a jet engine maintenance facility that operate all day round contribute to UFPs in this narrow wind sector. While their relative contribution to total UFPs present at night is sufficiently large to make a notable difference in the nucleation mode fraction (Figure 1f), absolute UFP concentrations remain low also in this wind sector (Figure 1c). In addition, early morning (between 05:00 and 06:00) road traffic emissions contributed to nucleation mode UFPs observed in this wind sector, as discussed later.

To further assess the contribution of the airport emissions to high UFP concentrations measured at the site, the diurnal cycle of size distributions, as well as the air traffic data, is plotted in Figure 2 for the wind directions between W and NE (247.5 to 67.5°, which are associated with the highest UFP concentrations; Figure S4 provides the same for all wind directions).

**Figure 2:**
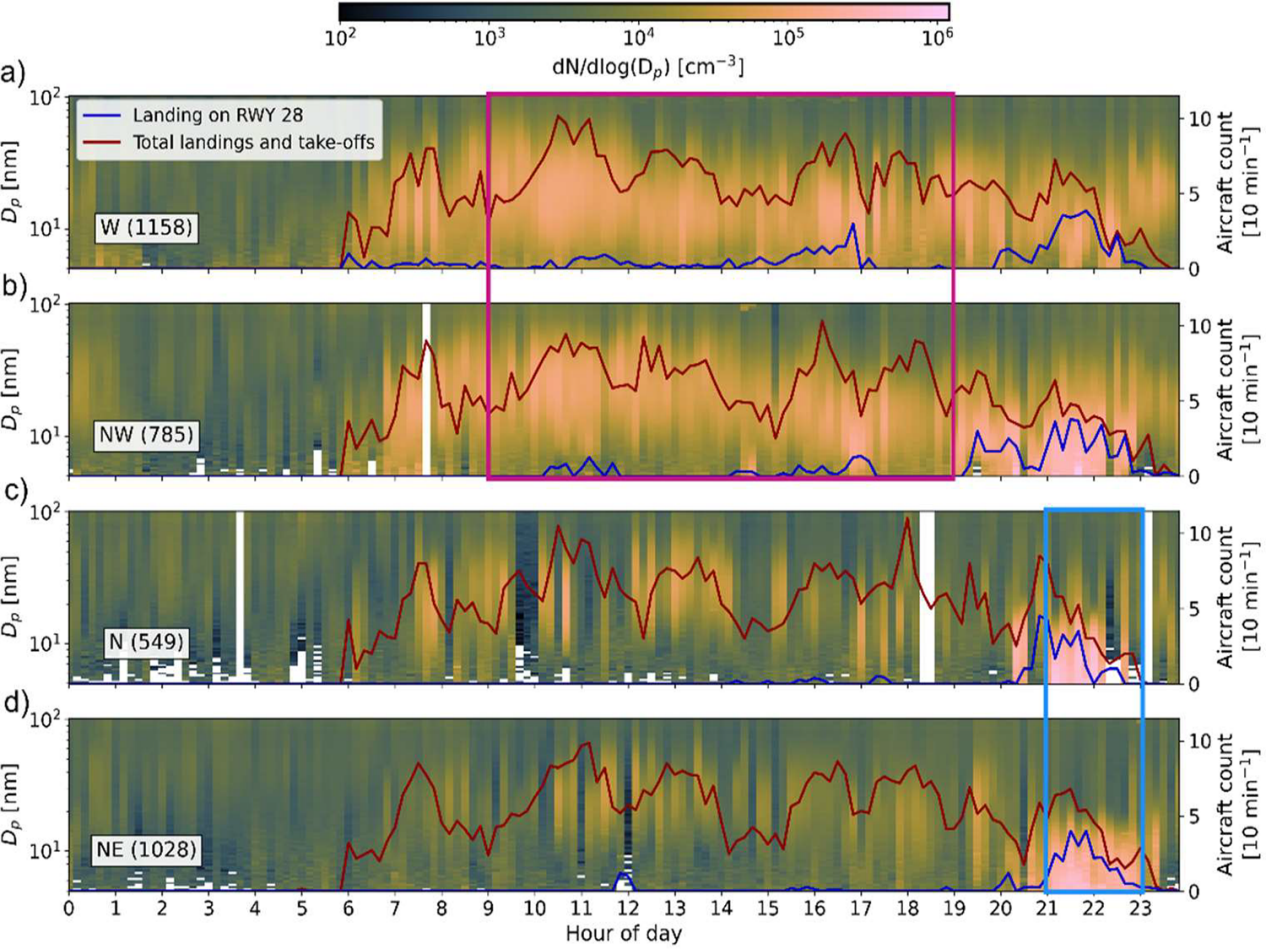
10 min averaged UFPs size distributions for (a) west (247.5–292.5°), (b) northwest (292.5–337.5°), (c) north (337.5–22.5°), and (d) northeast (22.5–67.5°) wind directions. Numbers in parentheses show the number of data points used to plot the panel. Red and blue lines show the sum of takeoffs and landings on all runways and the landings on runway 28 per 10 min, respectively. Colored frames indicate the data selected to extract the averaged size distributions represented in Figure 3a. For all wind sectors, see Figure S4.

For the downwind sector and during the entire air traffic operation hours, i.e., from 06:00 until 23:00, the UFP number concentration is strongly enhanced compared to nighttime (panels 2a and 2b) as also seen in Figure 1. About two-thirds (∼64% by number) of these UFPs fall into the nucleation mode size range with a diameter of <20 nm, while the other 36% fall into the diameter range from ∼20 to 80 nm, commonly named the Aïtken mode.^36,37^ UFP concentration enhancement in the downwind sector is most pronounced between 10:00 and 11:00 when air traffic is at a maximum, with an average of up to one flight per minute.

A distinct nucleation mode associated with high UFP concentrations appeared at around 21:00 when the wind was coming from the NW, N, and NE sectors (Figure 2b–d). This increase is due to the landings on runway 28. These aircraft are passing over the site as low as ∼80 m above ground, such that the wake dynamics result in subsidence and downmixing of the exhaust plume to the ground. Such plume subsidence by up to ∼300 m vertical descent is known to happen on a short time scale.^38^ The contribution of landing overpass to UFP concentrations observed at the site is not tied to downwind conditions, hence explaining the high UFP concentrations outside the downwind sector as seen in Figure 1c and the high nucleation mode fraction associated with it (Figure 1d).

Assigning the UFPs and variability of their properties as observed during daytime downwind conditions to engine aircraft operation, i.e., landing, taxi, or takeoff, is difficult. Neither the GMD nor the nvPM number fraction changed as a function of the prevalence of takeoff on landing movements (Figure S5), which suggests that the departure phase of the LTO cycle (engine start-up, taxiing, takeoff, and climb out) dominated the emissions during daytime. This hypothesis is in line with previous studies showing a higher contribution of 70% of the takeoff movements on the PN concentration compared to landings.^39,40^ Since these emissions are transported to the site by the wind in the same way, we were not able to distinguish between the phases making up the takeoff-related emissions, especially the taxiing emissions contribution. In addition, trace gas ratios, e.g., NO_*x*_/NO, were analyzed but did not provide insights on the relative contribution of the different engine loads (see Figure S6 showing the time series of the aerosol and trace gas measurements analyzed in this study, as well as the air traffic data). This is because the airport exerted a less pronounced influence on the concentrations and variability of these trace gases, in contrast with its dominant influence on UFPs. Previous studies reported that the taxiing duration time associated with departure is 2–2.5 times longer than the one associated with arrivals.^41,42^ Studies at other airports show that the low engine thrusts in taxi and running auxiliary power units (APU) can contribute significantly to UFP number concentrations.^43,44^ However, the Zurich Airport enforces strict regulations regarding the use of APUs. For example, it mandates the use of a fixed aircraft ground energy system at all gate parking stands and optimizes ground operations to minimize aircraft idling time.^45,46^ Measurement closer to the airport would be needed to clearly distinguish jet engine emissions from different operational phases.

To better visualize the difference between the daytime downwind and the evening size distributions linked to landing overpass, the size distributions of both total PM and nvPM averaged on data represented by the pink and blue frames in Figure 2 are plotted in Figure 3a. The nvPM size distributions were similar for observations dominated by the downwind and landing overpass plumes, with GMDs of 9.2 and 12.9 nm, respectively (Figure 3a). Previous test cell studies at the engine exit plane noticed an increase in nvPM GMDs with engine thrust.^9,47^ The above interpretation that emissions at low engine load (start-up and taxiing) also made a high contribution to the total ground emission of the departure phase of the LTO cycle likely explains the small difference we observed in nvPM GMD between the landing overpass and downwind plume. Two distinct modes, one in the nucleation range and one in the Aïtken range, can be observed at around 11–14 and 70–80 nm in the nvPM size distributions. To investigate their main contributing sources, the nvPM size distributions for morning downwind, morning not downwind, and nighttime are represented in Figure S7 on a linear *y*-axis scale. The morning window (from 07:00 to 10:00) was chosen when the airport activity had started well but early enough to avoid the effects of dilution when the boundary layer rose later in the day. The nucleation mode on the morning downwind curve has a 3-fold higher contribution to the number concentration than the Aïtken mode (gray shaded areas in Figure S7). Conversely, when no airport influence is expected (i.e., during the night and not downwind of the airport), the ratio between the nucleation mode and the Aïtken mode is around 1.4. This result shows that although emissions from the airport make the dominant contribution to the nucleation mode, the Aïtken mode mainly originates from a mix of background and road traffic emissions with a minor contribution from airport emissions.

**Figure 3:**
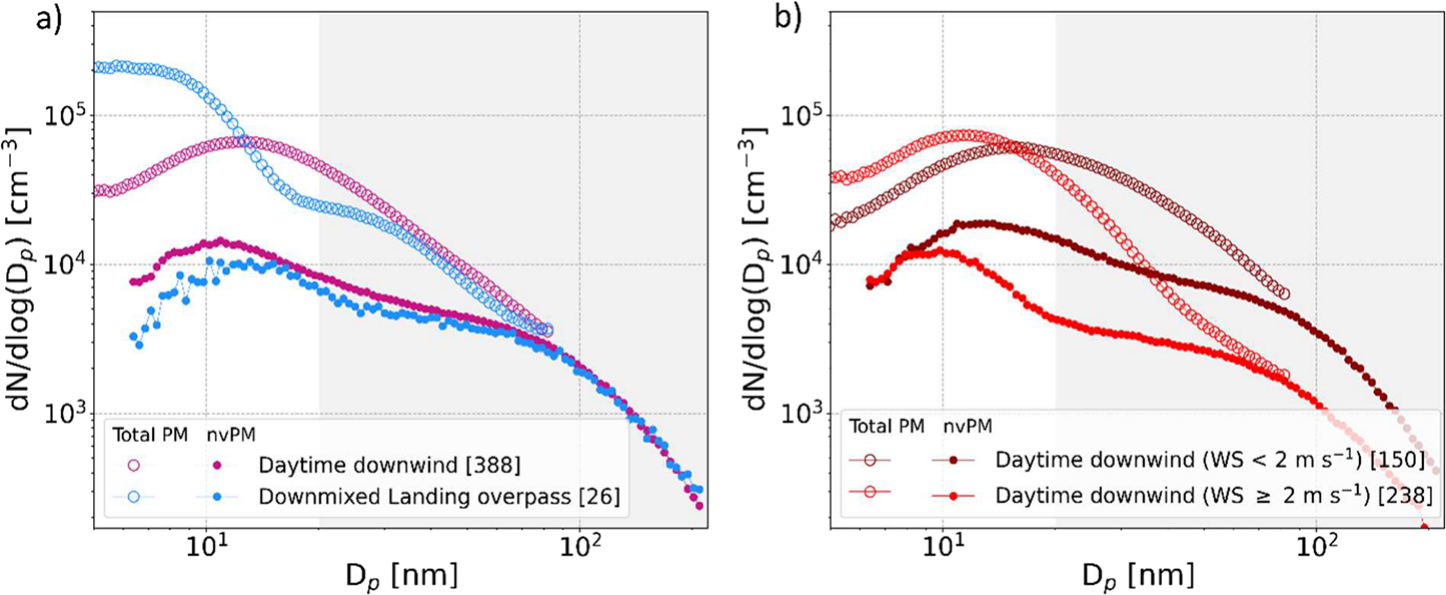
(a) Daytime downwind the airport and evening downmixed landing overpass and (b) Daytime downwind the airport with wind speeds < and ≥2 m s^–1^ averaged size distribution of total PM in open circles and nvPM in plain circles. Data selection was based on time ranges and wind directions represented with the colored frames in Figure 2. “Daytime downwind” is obtained when wind direction is from W to NW and outside of the road traffic rush hour peak (09:00 to 19:00) and “Downmixed landing overpass” is obtained when wind direction is from N to NE in the evening (21:00 to 23:00). The gray shaded areas (white) represent the corresponding diameter ranges for which the dominant source is a mix of background and road traffic emissions with a minor contribution from airport emissions (dominated by airport emissions). A copy of the nvPM size distributions shown in this figure is provided in Figure S7b,c, which use linear instead of logarithmic ordinate scaling.

The main difference between the two total PM size distributions lies in a dominant nucleation mode for landing overpass, with a GMD as small as 6.9 nm and concentrations 4 times higher than during daytime downwind periods. Engine load during approach is on average low, which comes with enhanced emission factors of semivolatile vapors. This favors new particle formation through homogeneous nucleation over condensation onto nvPM particles as the exhaust plume cools during dilution with ambient air, thus resulting in a dominant nucleation mode and high vPM number fraction (87.9%). UFPs observed in the downwind sector are also dominated by vPM (75.5%) and small GMD (∼13.8 nm). The downwind plume also has a large contribution from emissions at a low engine load. However, the transport time from the emission to the site is longer, thus potentially allowing for more condensation and coagulation. Formation of vPM in the nucleation mode size range driven by dilution cooling has previously been shown. For example, Beyersdorf et al.^48^ reported a dominant nucleation mode 145 m behind the aircraft that was not present at the engine exit plane at lower thrusts (4–65% thrust). Hudda et al.^2^ reported PN concentrations (lower cutoff at 7 nm in diameter) more than 7 times higher for overhead landings compared to takeoff near the Logan International Airport, Boston. Consistent with this, we also found a decrease of GMD along with a concurrent increase of vPM number fraction for daytime observations in the downwind sector at times when the landing fraction of all aircraft movements increased to 83% or higher (i.e., less than 17% takeoff), which is seen in Figure S5. The results in Figure 3b, which will be discussed below, further show that coagulation occurring in the downwind plume between the airport and the Kloten site can make a notable shift of the size distributions to larger diameters. This study highlights that near the source, the high fraction of vPM is a distinct feature of aircraft emissions.

To assess the relative importance of road and air traffic for air quality in Kloten, a statistical analysis of the UFP diurnal cycle has been carried out using 2022–2023 data. A selection of quantiles of *n*_totalPM_ covering the range from the 90th to the 10th percentile is determined for different subsets of all data, i.e., separately for weekdays versus weekends and for all wind sectors versus the downwind sector (see Figure 4). The UFP concentration level exceeds 30,000 cm^–3^ in around 30% of all daytime data (i.e., for percentile *X* = 70, see Figure 4b), but for <10% of data points when Kloten is neither downwind nor affected by the landing overpass (in the evening) (Figure S9s). For data exceeding the 30,000 cm^–3^ threshold, the difference between weekday and weekend diurnals is negligible. Below 30,000 cm^–3^, the concentration level on weekdays often exceeds that of the weekend. More precisely, this difference is seen for all daytime data when considering all wind directions (Figure 4c–e), whereas it is only seen during the early morning rush hour for the downwind sector (Figures 4h,j and S9). This analysis provides two results: first, traffic emissions do make a substantial contribution to UFPs whenever UFP levels are low to medium. Second, at times when the UFP levels are low to medium, the airport emissions still make a substantial contribution to UFPs if the Kloten site is downwind of the airport. Variations of the UFP concentration observed for downwind conditions are likely driven by horizontal wind speed and vertical mixing. It is important to note that percentiles reported in the left-hand side panels of Figure 4 depend on the angle values chosen to represent the downwind sector. Further, the large difference between the mean and median for all data relative to downwind data (Figure 4c,h) indicates asymmetry of the UFP concentration frequency distribution due to short but strong airport-caused concentration spikes.

**Figure 4:**
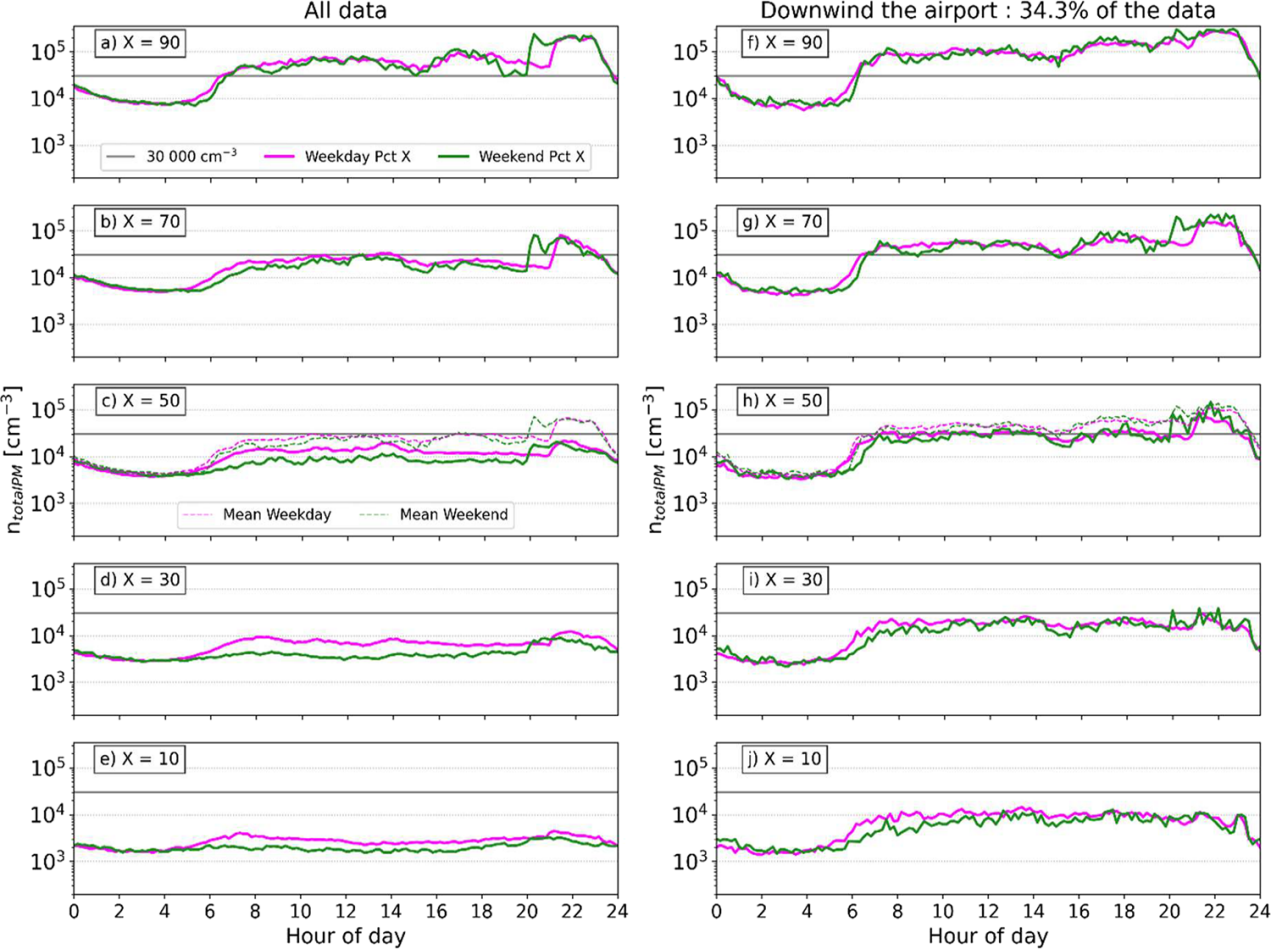
From top to bottom: 10 min averaged daily cycle of UFP number concentration from the long-term (2022–2023 included) data set for different decreasing percentiles, from 90th to 10th, for all data and downwind data. For each panel, weekday and weekend diurnal cycles are represented in pink and green lines, respectively. In addition, on the 50th percentile panels (c,h), the mean values are plotted in dashed lines, with the same color code as the plain lines. A horizontal gray line on every panel represents a UFP number concentration of 30,000 cm^–3^. See Figure S9 for the detailed intermediate percentiles and not the downwind data.

An additional influence of UFPs downmixed from the landing overpass is clearly discernible in Figure 4 as a step increase of UFP concentrations at 21:00 or 20:00 during weekdays or weekends, respectively, in line with the times when landings switch to runway 28 under the most weather conditions due to the special regulations of the ZRH airport (see Figure S10 showing the diurnal pattern of air traffic). This landing overpass influence, highlighted previously, increases *n*_totalPM_ above 30,000 cm^–3^ around 50% of the time in the evening time window (even 70% for downwind conditions and still 40% outside the downwind sector). This evening structure remains discernible down to the 20th percentile (Figure S9h), i.e., 80% of the time, showing the great influence of landing overpass emissions on air quality at the Kloten site.

Most of the particles from the airport emissions are in nucleation mode (see above and Figure 3a). To further assess UFPs in the nucleation mode (diameter <20 nm), we provide a detailed statistical analysis of their diurnal pattern in Figure S11. Overall, the result is qualitatively consistent with the result for total UFPs (Figure S9), but for lower concentrations, when only considering the nucleation mode (Figure S11). This difference is important and surprising because it shows that the airport emissions also increase UFP concentrations in the Aïtken mode, a finding which will be addressed in more detail in the following subsection.

Figure S11 further reveals clear differences between weekdays and weekends at times when UFPs are not dominated by airport emissions. This clearly shows that road traffic contributes substantially to the nucleation mode, particularly during the early morning. This agrees with previous studies highlighting an important contribution of road traffic on nucleation mode particles.^47–49^

### UFP Diameter and Volatile Fraction Can Rapidly Change in the Near-Field Plume

The GMD of the UFP size distribution in the downwind airport plume was 13.8 nm on average (Figure 3a) and smaller than 20 nm for more than 75% of the 10 min data (Figure S5). However, occasionally the GMD increases substantially above 20 nm, for example, during the 5 episodes with high SO_2_ concentrations from October 27th to 31st (Figure S6).

The analyses presented in Figure 5 provide evidence that certain meteorological conditions facilitate the growth of UFPs to the Aïtken mode during transport from emission to the receptor site. For wind speeds >2 m s^–1^, the GMD of total PM always remains below 20 nm when dominated by airport influence (Figure 5a). By contrast, GMDs between 20 and 40 nm frequently occur for wind speeds below 2 m s^–1^. Similarly, the GMD of nvPM also systematically increases at these low wind speeds up to a maximum of ∼20 nm (Figure 5b), which is the result of a systematic shift of all nvPM particles in the dominant mode to bigger sizes (Figures 3b and S7c). This increase in the nvPM GMD provides clear evidence of coagulation happening during transport of the aviation emissions to the site. The number fraction of nvPM increases from values below 40% at wind speeds >2 m s^–1^ to values reaching up to 95% at lower wind speeds. This is also consistent with coagulation, as the collision of one vPM particle with one nvPM particle results in one nvPM particle, thus increasing the nvPM number fraction. Lower wind speed increases transport time from emission to the site, thus leaving more time for coagulation to have a notable effect on GMD. Lower wind speed also means that the emissions at the airport are injected into a smaller air volume passing by the airport, thus quenching dilution when plumes from multiple aircraft mix with each other. This results in higher concentrations of all gaseous and particulate pollutants. Indeed, the highest SO_2_ concentrations, which are an inversely related indicator of dilution of the aviation emissions, occur at low wind speeds (color code of data points in Figure 5a–c). More so, increased SO_2_ concentrations coincide with increased GMD and an increased nvPM number fraction. This is particularly the case for the period between October 27th and 31st, showing the growth of UFPs GMD up to 40 nm, due to stable wind speed conditions associated with low dilution of the aviation emissions reflected in SO_2_ concentrations as high as 6 μg m^–3^ (Figure S6). Reduced dilution at low wind speed, which has a squared effect on coagulation, may outrun the effect of increased transport time, which has only a linear effect. The data presented in Figure 5 only covers a limited observation period of 29 days. The same analysis as presented in Figure 5a has been carried out on the long-term data from the Kloten site and confirms that this finding is of general importance for the evolution of the UFP size distribution in the airport plume (Figure S13). Therefore, we conclude that below-average dilution of the airport emissions, which can occur at low wind speeds, drives growth of the UFPs from the nucleation mode into the Aïtken mode during transport to the measurement site through enhanced coagulation caused by higher concentrations and longer transport time. Higher concentrations of condensable vapors under these conditions may also result in stronger condensation growth of the UFPs. However, our data does not allow us to quantify this additional effect. To sum up, we found that UFPs from aviation emissions can undergo growth by coagulation and condensation even over relatively short transport distances of roughly 1 km. This agrees with previous studies that reported GMDs > 20 nm downwind from the airport and at background sites.^50–54^

**Figure 5:**
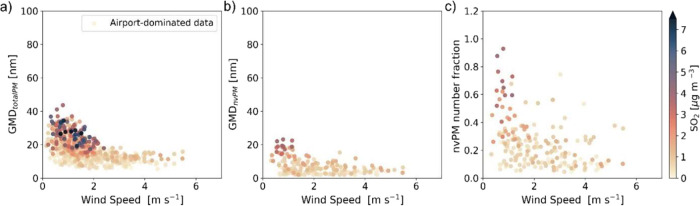
(a) Total PM GMD, (b) nvPM GMD, and (c) nvPM number fraction of airport-dominated UFPs, defined as UFP number concentrations >30,000 cm^–3^, as a function of the wind speed for the whole measurement campaign. Panel a is plotted for the entire measurement campaign (from October 25 to December 3), while panels (b) and (c) show the nvPM data covering the period from November 16 to November 29. The 10 min averaged data are colored by SO_2_ concentration, which serves as an indicator of the aviation emissions dilution. To exclude the case of landing overpass, only downwind data before 19:00 were accounted for in the airport-dominated data. The same figure with all data points is shown in Figure S13.

### Traces of Aircraft Engine Lubrication Oil during Airport-Influenced Events

Previous work^18^ has shown the uniqueness of jet engine lubrication oil compounds, e.g., synthetic ester homologues and TCP, as aviation tracers. Using engine test cell measurements, Decker et al.^33^ demonstrated that the EESI-LToF-MS is capable of detecting these tracers in engine emissions. Here we investigate the potential of this technique to use these oil compounds as tracers for aviation emissions at the Kloten field site.

For this purpose, we present the PN concentration measured by the CPC3756 as a function of the measured EESI-LToF-MS signal for measured ions corresponding to oil tracers (Figure 6a–d) and nonaircraft sources (Figure 6e–h). The 1 min data of the CPC3756 were aggregated to box and whisker plots of PN number concentration for EESI-LToF-MS signal intensity ranges covering 0–1, 1–2, 2–3, and >3 times the standard deviation of the background signal (σ_bck_ on the *x*-axis). For the top four panels, the following lubrication oil markers were used to group the PN concentration data: (a) TCP, (b) C_25_, (c) C_27_ PA esters from Mobil Jet II oil, and (d) C_27_ TMP ester from BP engine oils (see Table S1 for more details on these compounds). Considering TCP, C25 Mobil, and C27 Mobil in Figure 6a–c, the PN concentrations increased by a factor of 2.5–3 as the EESI-LToF-MS signal intensity increased from 1 σ_bck_ to 3 σ_bck_. This demonstrates that oil compound concentrations reflect the strength of the airport influence, which has been shown to be the main driver of the UFP enhancements. The signal-to-noise ratio for the oil compounds was not sufficient to use them as tracers on the level of individual 1 min data points; however, overall, it is a robust predictor: the higher intensity signals of TCP above 2 σ_bck_ were mainly found when the site was either influenced by the downwind airport plume during the day or by the downmixed plumes from landing overpass (Figure S14). By contrast, the TCP signals below or at the detection limit (<1 σ_bck_) were mostly measured outside operation hours, outside the downwind sector, or when PN concentration was low. Similarly, for the oil compounds C25 and C27 Mobil, being downwind of the airport was associated with a larger fraction of high signal intensities compared to being outside the downwind sector (Figure S15a–c). In contrast to the studied Mobil Jet Oil II compounds (Figure 6a–c), no clear correlation between the PN concentration and signal intensities for C27 BP has been found (Figure 6d). Indeed, it can be expected that Mobil Jet Oil II emissions prevail over BP oil emissions as Swiss International Air Lines, which makes up more than half of the air traffic at Zurich Airport,^55^ exclusively uses Mobil jet Oil II (source: private communication).

**Figure 6:**
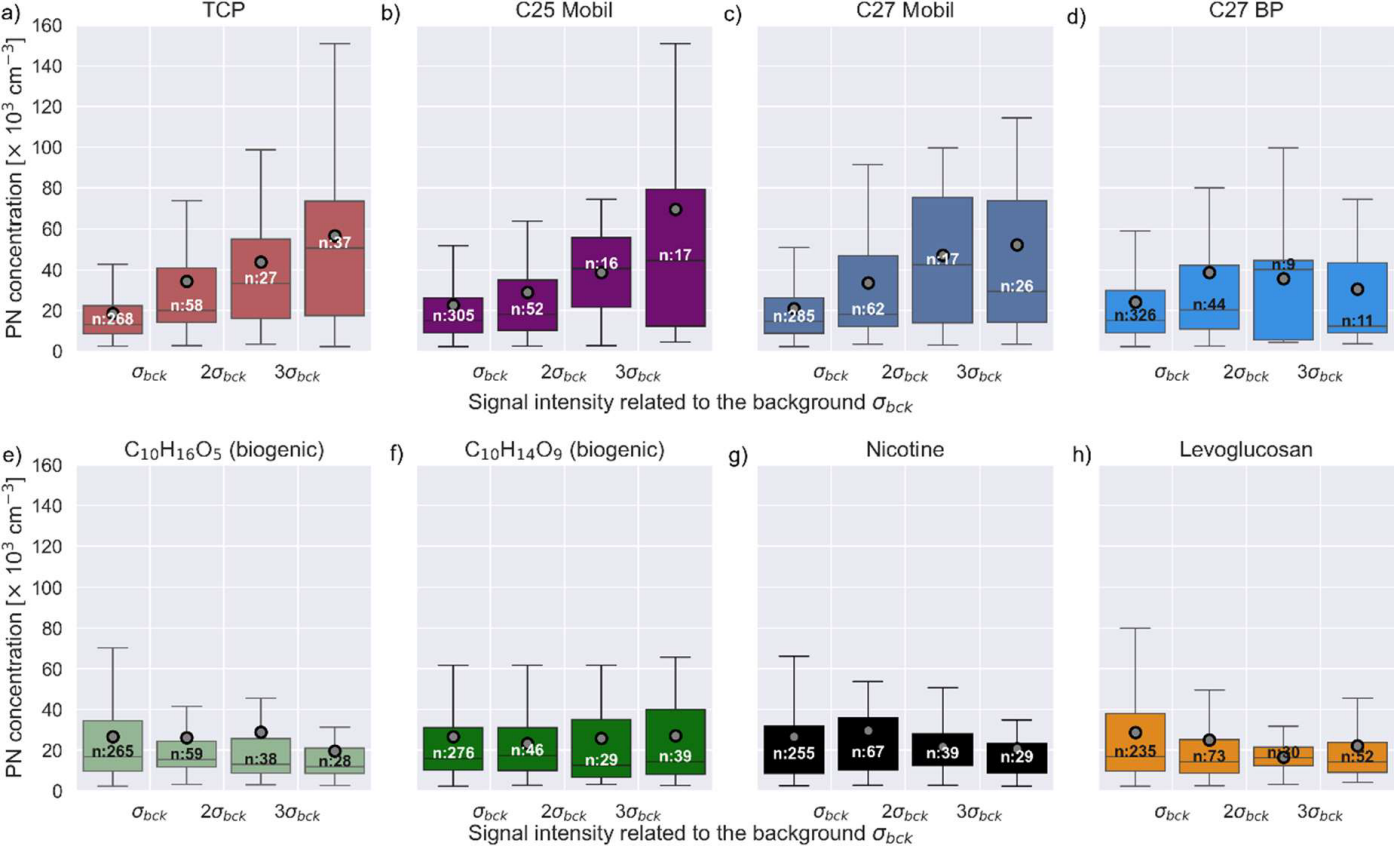
Particle number concentration from the CPC as a function of the EESI-LToF-MS signal intensities for different compounds. Signal intensities have been grouped related to one, two, or three times the standard deviation value σ_bck_ of the component background measurement. Boxes, lines, gray dots, and whiskers indicate the 25th percentile and 75th percentile, median, mean, and 10th percentile and 90th percentile, respectively. Numbers on the boxes are the number of data considered. Only daytime data (from 06:00 to 19:00) with wind speeds of >1 m s^–1^ were considered. Data is 1 min averaged.

As a negative control, the PN concentrations were also plotted against signal intensities of two biogenic compounds from α-pinene oxidation products (C_10_H_16_O_5_ and C_10_H_14_O_9_), nicotine and levoglucosan, which are tracers for smoking and biomass burning emissions, respectively (Figure 6e–h). As expected, no correlation has been found for these compounds since neither biogenic nor biomass burning emissions drive high PN concentrations at the Kloten site. This corroborates the causal relationship between the presence of oil compounds and high PN concentrations from aviation.

The oil compound measurements are neither size-resolved nor in calibrated concentration units. Therefore, we cannot quantify the contribution of oil compounds to the total UFP mass. Engine test cell measurements suggest both UFPs and particles between 0.1 and 1 μm likely contain significant oil mass.^33^ Nonetheless, results from a previous study at Frankfurt Airport indicate that the oil mass fraction can be high in small UFPs (from 10 to 18 nm).^18^

### Implications

Our measurements show that UFP concentrations up to 300,000 cm^–3^ originating uniquely from airport operations are commonly observed in this residential area 1 km downwind of Zurich Airport. The highest concentrations were observed when the downmixing of the exhaust plume from aircraft passing directly over the site during approach additionally contributed to UFP immissions. Through simultaneous online aerosol mass spectrometry, we further demonstrated that jet engine lubrication oil compounds can serve as unique tracers for the influence of aircraft emissions. Wider application of this online method to assess exposure to oil compounds at larger distances and with high time resolution would require further refinement of the analytical methods.

When the site was downwind of the airport, the hourly averaged UFP number concentration exceeded the WHO lower threshold for ”high PN concentrations”^56^ about 60% of the time (see Figure S16), thus making it mandatory to monitor, in accordance with the revised European Ambient Air Quality directive published in 2024.^57^

Considering that more than 200,000 inhabitants are living in the surrounding area of Zurich Airport,^58^ the high UFP number concentrations and the associated presence of aircraft lubrication oil are a potential public health concern.^59–61^ While previous studies suggest that the more toxic ortho-substituted TCP isomers are no longer detectable in aircraft lubrication oils,^62^ our results demonstrate a widespread presence of potentially other TCP isomeric forms in aerosols transported into surrounding communities near Zurich Airport. These oil emissions should be technically addressed by engine manufacturers and should be part of future engine emission regulations.

## Supplementary Material



## Notes

Measurement site’s location and wind data; CPC data check and correction, list of EESI-LToF-MS analyzed ions; diurnal cycle of UFPs size distribution and air traffic; total PM GMD and nvPM number fraction variability as a function of takeoff prevalence, time series of wind data, air traffic, UFPs properties and gas measurements; nvPM size distribution for different emission influences; Diurnal cycle of UFPs number concentration, air traffic and road traffic; UFPs properties as a function of wind speed for all data and airport-dominated data; UFPs GMD as a function of wind speed for the long-term data set; occurrence of TCP signal intensities as a function of wind and airport influence; Ion signal intensities measured by the EESI-LToF-MS as a function of wind influence; long-term daily cycle of UFPs concentrations related to the WHO guidelines (PDF)

## §

NOAA CSL & Cooperative Institute for Research in Environmental Sciences (CIRES), Boulder, Colorado 80309, United States

## ∥

XRnanotech AG, Villigen 5234, Switzerland.
