# Supplementary material for: Ubiquity of Aviation Ultrafine Particles and Lubrication Oil Compounds Near Zurich Airport
Source: Environ Sci Technol. 2026 Apr 23;60(17):13051–62. doi: 10.1021/acs.est.5c18458 (PMC13151064; doi:10.1021/acs.est.5c18458)
Supplement: Supplementary file 1 [file es5c18458_si_001.pdf]

Supporting information for:

The Ubiquity of Aviation Ultrafine Particles and Lubrication Oil Compounds near Zurich Airport

Sarah Tinorua[1], Benjamin. T. Brem[1*], Zachary C.J. Decker[1,a], Jay G. Slowik[1], Peter A. Alpert[1,b], Markus Ammann[1], André S. H. Prévôt[1], Michael Bauer[1], Suneeti Mishra[1], Michael Götsch[2], Joerg Sintermann[2] and Martin Gysel-Beer[1]*

[1] PSI Center for Energy and Environmental Sciences, Villigen PSI, 5232, Switzerland

[2] Canton of Zurich, AWEL - Amt für Abfall, Wasser, Energie und Luft, Zurich, 8090, Switzerland

[a] now at: NOAA CSL & Cooperative Institute for Research in Environmental Sciences (CIRES), Boulder, Colorado, 80309, USA

[b] now at: XRnanotech AG, Villigen, 5234, Switzerland

*Corresponding Authors : Sarah Tinorua (**Sarah.tinorua@psi.ch**) and Benjamin T. Brem (**Benjamin.brem@psi.ch**)

Number of pages: 15

Number of figures: 16

Number of tables: 2

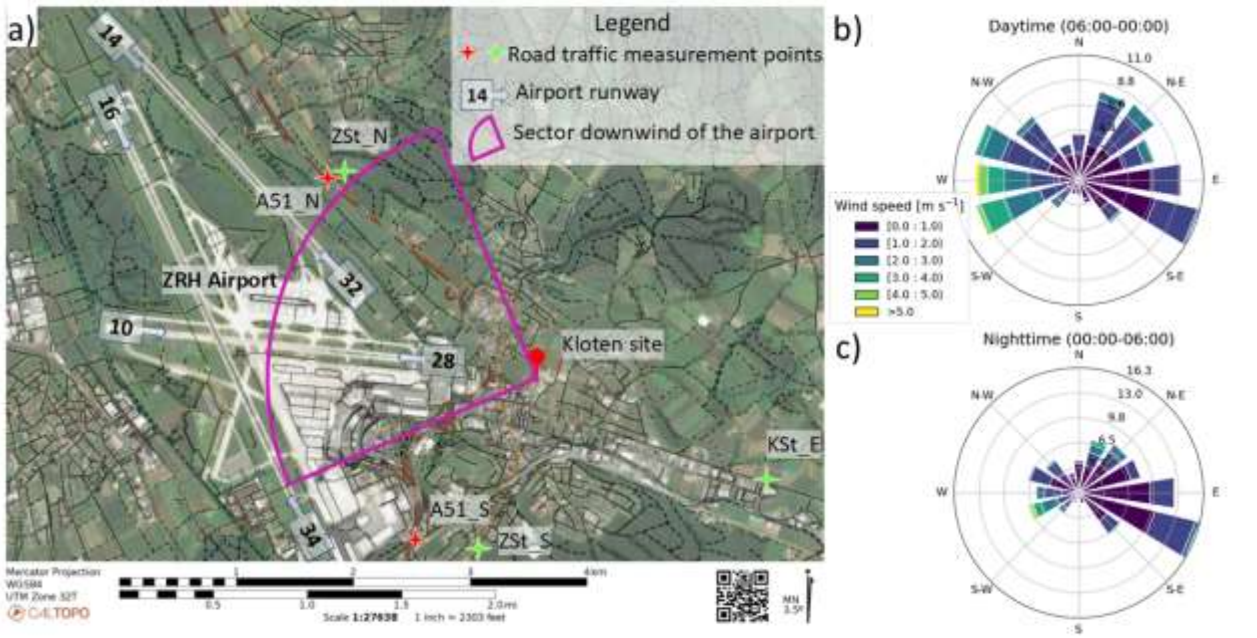

Figure S1: Kloten measurement site with the sector used for "downwind" data represented in purple. This sector was chosen wide enough to include the runway 34/16 and the beginning of the runway 32. Maps generated from CalTopo.com, reproduced with permission. Map data from OpenStreetMap and Sentinel-2 cloudless - https://s2maps.eu by EOX IT Services GmbH (Contains modified Copernicus Sentinel data 2019).

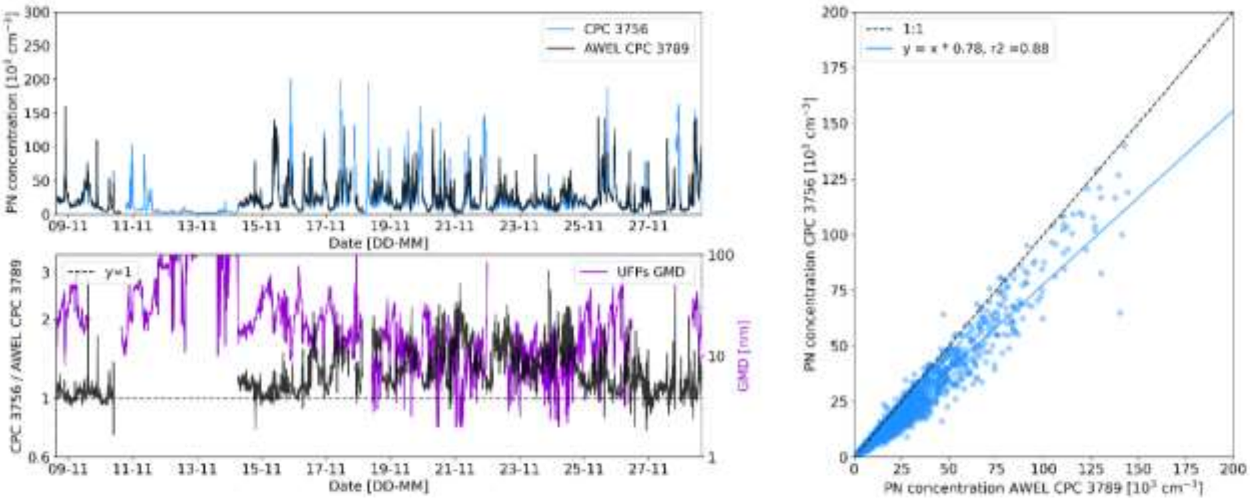

Figure S2: 10-minutes data comparison between the TSI CPC 3756 used in this study for PN concentration and the TSI CPC 3789 from the AWEL container. Both CPCs didn't have a sample dilution system. 10 min intervals with readings exceeding the the upper limit specified by the manufacturer (200'000 cm^{-3} for the AWEL CPC 3789 and 300'000 cm^{-3} for the TSI 3756) were not considered for this figure. The agreement between the two CPCs is on average within 22%, and the ratio between 0.7 and 3. We attributed this discrepancy to diffusion losses, that were accounted for only in the CPC 3789 data, and to the different sampling conditions (sampling flows, tube and inlet lengths). The increasing ratio between the two CPCs with decreasing GMDs (that causes increasing diffusion losses), for instance on November 21th and 24th , is supporting this.

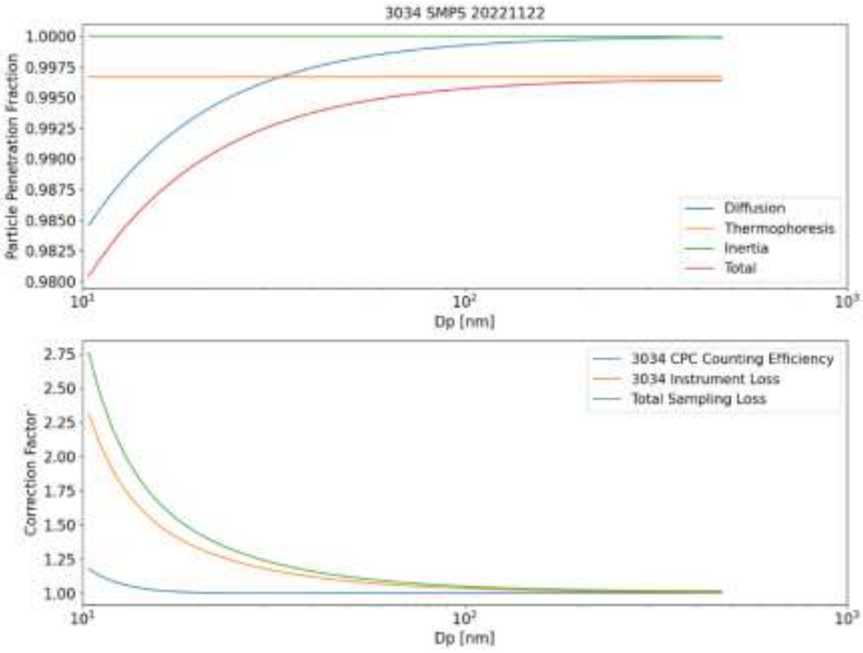

Figure S3: Particle penetration fractions and corresponding corrector factors applied to the SMPS3938 data as a function of the particle diameter. For each measurement day, one correction factor was calculated and applied to the measured size distributions. As particle penetration is also depending on the ambient temperature and the pressure, these figures show an example for November 22nd 2022.

Compound	Formula	m/z	Notation	Comment
Tricresyl Phosphate	$C_{21}H_{21}O_4PNa^+$	391.107	TCP	Present in jet engine oils and exhausts [1]
C25 PA Ester	$C_{25}H_{44}O_8Na^+$	495.293	C25 Mobil	Present in Mobil jet engine oils and exhausts [1]
C27 TMP Ester	$C_{27}H_{50}O_6Na^+$	493.350	C27 BP	Present in BP jet engine oils and exhausts [1]
C27 PA Ester	$C_{27}H_{48}O_8Na^+$	523.324	C27 Mobil	Present in Mobil jet engine oils and exhausts [1]
α-Pinene hydroxy dihydroperoxide	$C_{10}H_{16}O_5Na^+$	239.089	$C_{10}H_{16}O_5$	Present in α-pinene derived SOA; Tracer for biogenic PM [2]
Alpha-pinene SOA	$C_{10}H_{14}O_9Na^+$	301.053	$C_{10}H_{14}O_9$	Present in α-pinene derived SOA; Tracer for biogenic PM [2]
Nicotine	$C_{10}H_{14}N_2Na^+$	163.123	Nicotine	Tracer for anthropogenic emissions [2]
Levoglucosan	$C_6H_{10}O_5Na^+$	185.042	Levoglucosan	Tracer for biomass burning [2]

Table S1: List of the targeted compounds for the EESI-MS molecular analysis. Note that the m/z is the molecular weight of the analyzed compound associated with a sodium ion Na+, present in the electrospray solution (here a 50/50 Acetonitrile/water solution doped with sodium iodine NaI, as recommended by Bell et al. (2023)[3]).

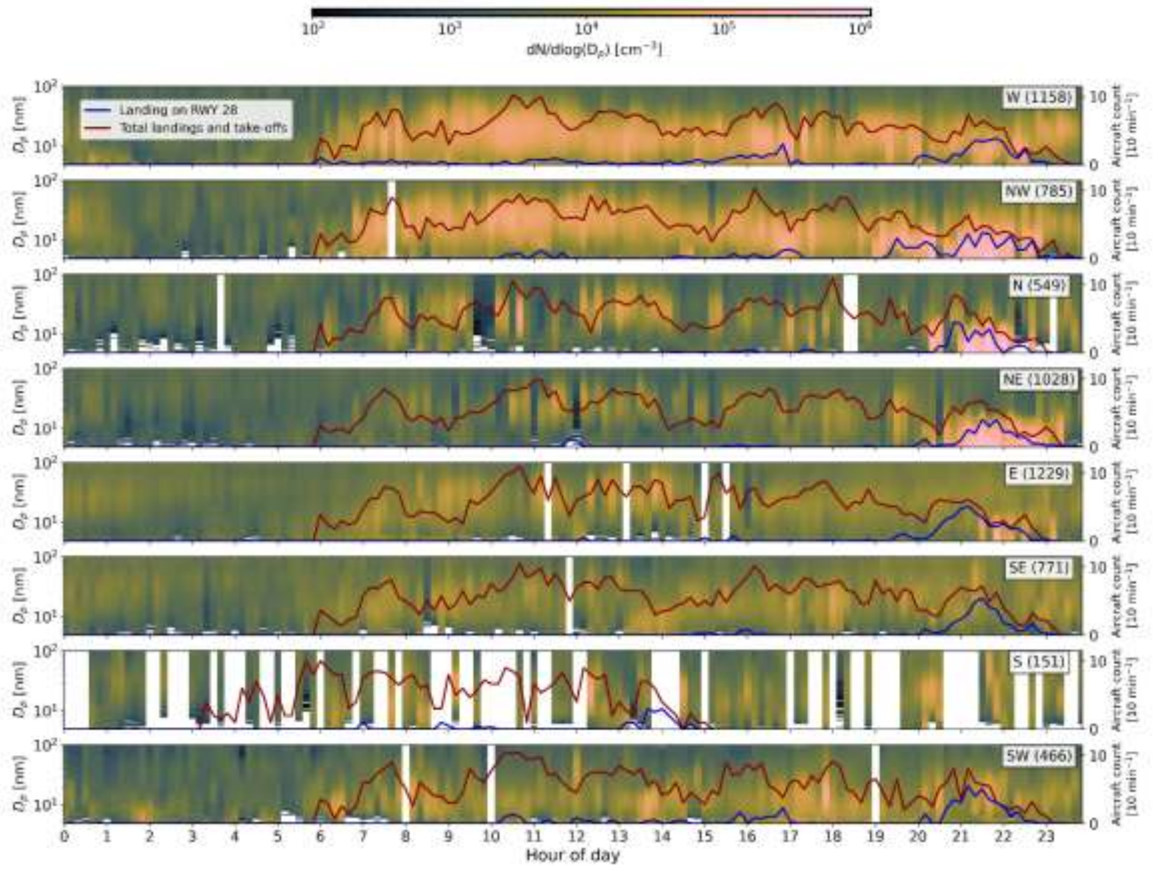

Figure S4: 10-minutes averaged UFPs size distributions averaged by wind directions from West to South-West. Numbers in parenthesis show the number of data-points used to plot the panel. Red and blue lines show the sum of take-offs and landings on all runways, and the landings on runway 28 per 10 minutes, respectively. The highest UFPs concentrations during the day were measured downwind the airport (W and NW wind directions), while during the evening between 19:00 and 23:00 they were coming from the NW to NE, due to the aircraft landing over the site from NE.

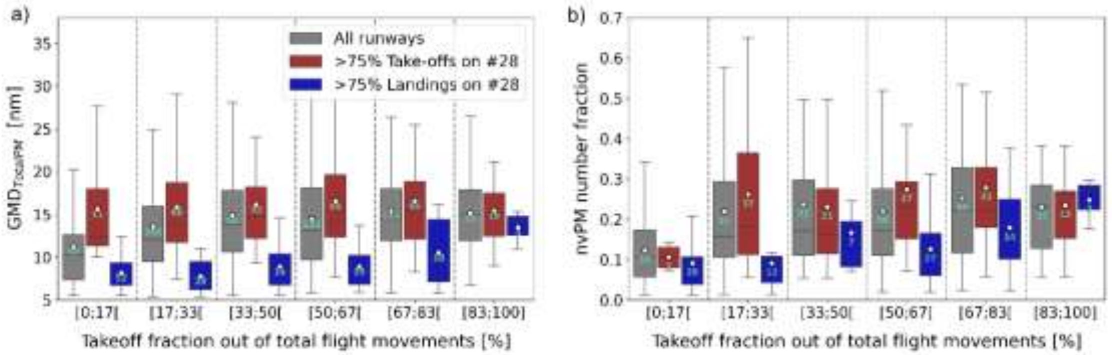

Figure S5: Box and whisker plots of a) fitted GMD of total PM number size distribution and b) nvPM number fraction as a function of take-off fraction of total aircraft movements. Boxes, lines, grey dots and whiskers indicate the 25th percentile and 75th percentile, median, mean, and 10th percentile and 90th percentiles of the 10-minutes averaged data, respectively. The grey boxes represent the data for all the runways, while the colored boxes show only the data for which more than 75 % of the movements occurred on runway 28, for which aircraft flew over the measurement site. Both the GMD and the nvPM number fraction were constant with the take-off fraction, indicating that emissions associated with the departure phase are dominating the airport related UFPs and thus their properties during daytime.

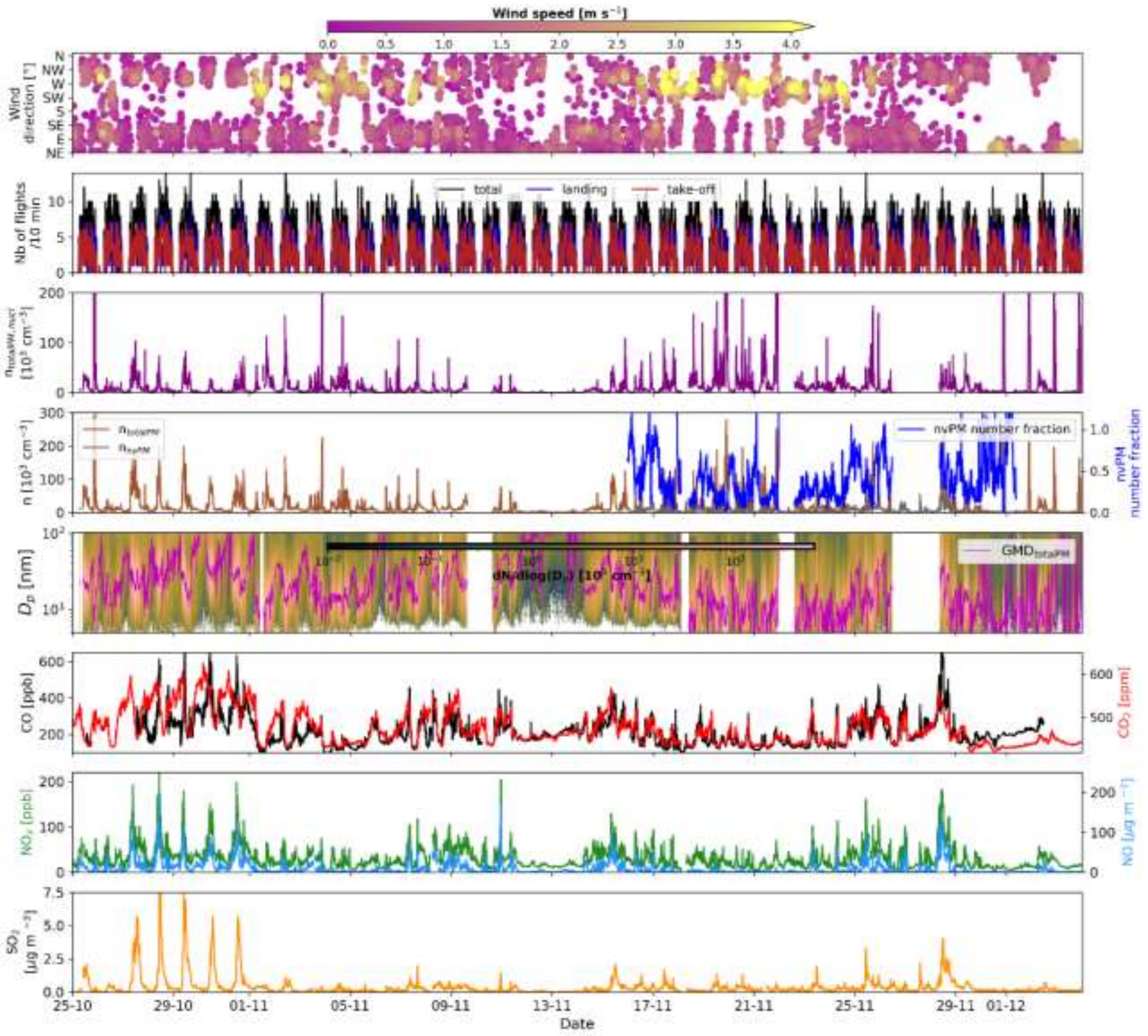

Figure S6: Time series of wind direction and speed, air traffic at Zurich Airport, number concentrations of the UFPs in the nucleation mode ($n_{totalPM,nucl}$), the total PM ($n_{totalPM}$) and nvPM (n_{nvPM}), as well as the nvPM number fraction, UFPs size distribution, CO, CO_2 and NO_x mixing ratios, and, NO and SO_2 mass concentrations measured over the autumn 2022 at Kloten. Very high total PM number concentrations above 100'000cm^{-3} were measured at Kloten, together with peaks of high combustion gases. The simultaneous increase of UFPs number concentrations in the nucleation mode reveals its significant contribution to the total PM. These high pollution episodes seem to come from the W-NW direction. The last five days of October were characterized by particularly high combustion gases peaks, and higher GMDs of the UFPs, which have been shown to be due to condensation/coagulation processes.

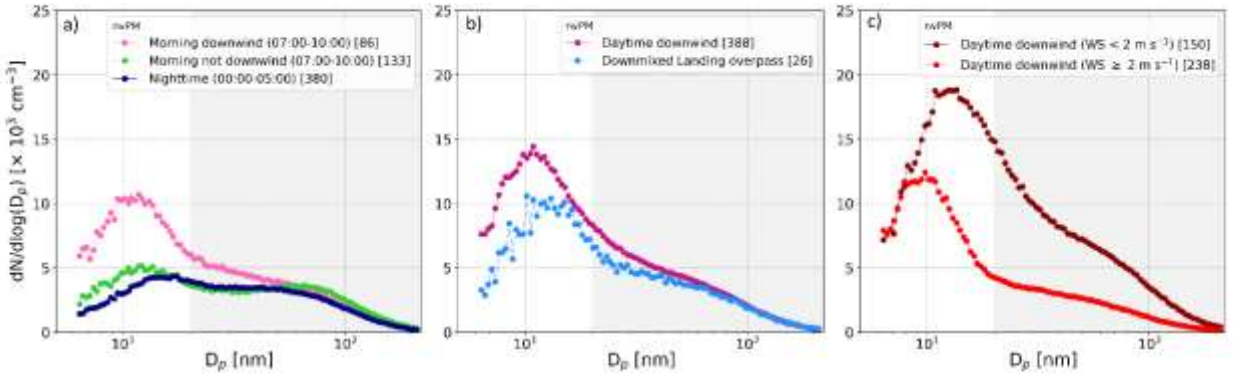

Figure S7: a) Morning downwind, not downwind the airport, and nighttime averaged size distribution of nvPM. Panels b) and c) are the same panels as Figure 3a and b but using a linear y-axis scale. "Morning downwind" (resp. not downwind) is obtained when wind direction is from W to NW (resp. when wind direction is not from W to NW) and between 07:00 and 10:00. "Daytime downwind" (resp. not downwind) is obtained when wind direction is from W to NW (resp. when wind direction is not from W to NW) and outside of the road traffic rush hour peak (09:00 to 19:00). "Nighttime" is obtained between 00:00 and 05:00. While "morning downwind" is expected to be dominated by airport emissions, "morning not downwind" and "nighttime" are expected to reflect mixed background and road traffic influence. The grey shaded areas (resp. white) represent the corresponding diameter ranges for which the dominant source is a mix of background and road traffic emissions with a minor contribution from airport emissions (resp. dominated by airport emissions).

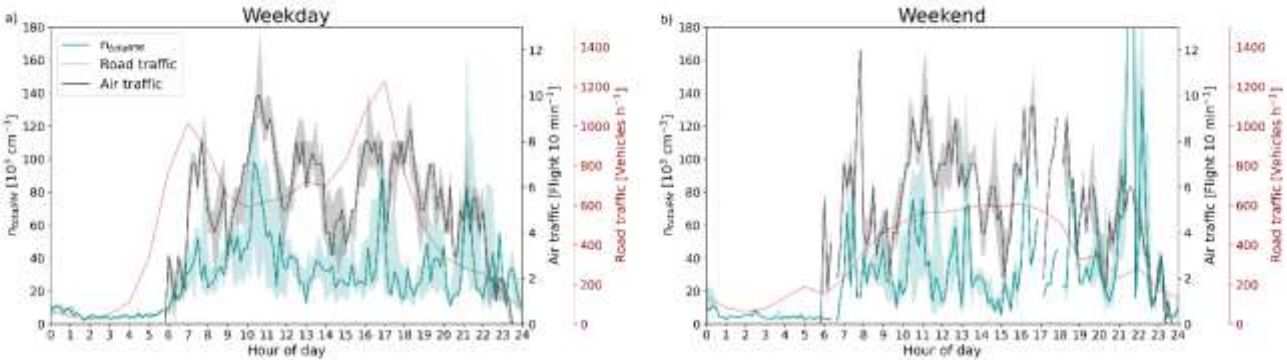

Figure S8: a) Weekdays and b) weekends diurnal cycle of air traffic at Zurich Airport, road traffic, and UFPs number concentration $n_{totalPM}$ when Kloten site is downwind the airport, i.e. when the wind direction is comprised between West and North-West. Road traffic has been calculated using the western counting points, i.e. the points labelled as ZSt_N, A51_N and A51_S on Fig. S1. Plain lines and light shaded areas for air traffic and $n_{totalPM}$ represent the median and the interquartile ranges of the 10-minutes averaged data, respectively. Similarities between the UFPs concentrations and the air traffic movements, especially on weekdays show the influence of air traffic on the site. However, the poor statistics of the data during the weekend didn't allow us to investigate the contribution of road traffic.

Comment on Figure S8:

To assess the impact of road traffic compared to air traffic on UFP concentrations, its diurnal cycle, as well as air and road traffic have been investigated in Fig. S8. As opposite to weekdays, the road traffic during the weekend daytime is rather constant. Therefore, we provide the diurnal patterns separately for weekdays and weekends, to avoid weakening potential morning rush hour effects by averaging with weekend days. Daytime UFP concentration levels exceed nighttime concentration levels by more than an order in magnitude, thus clearly pointing to local or regional anthropogenic sources. Several features in the diurnal pattern provide clear evidence that this increase is driven by aviation emissions rather than traffic. The sharp increase related to evening landing overpass is clearly visible in both weekdays and weekends diurnal cycles. On weekdays, the daytime UFP levels are relatively constant, similar to a rather constant air traffic frequency. By contrast, the pronounced morning rush hour peak in road traffic occurring on weekdays is not reflected in a concurrent UFP concentration peak.

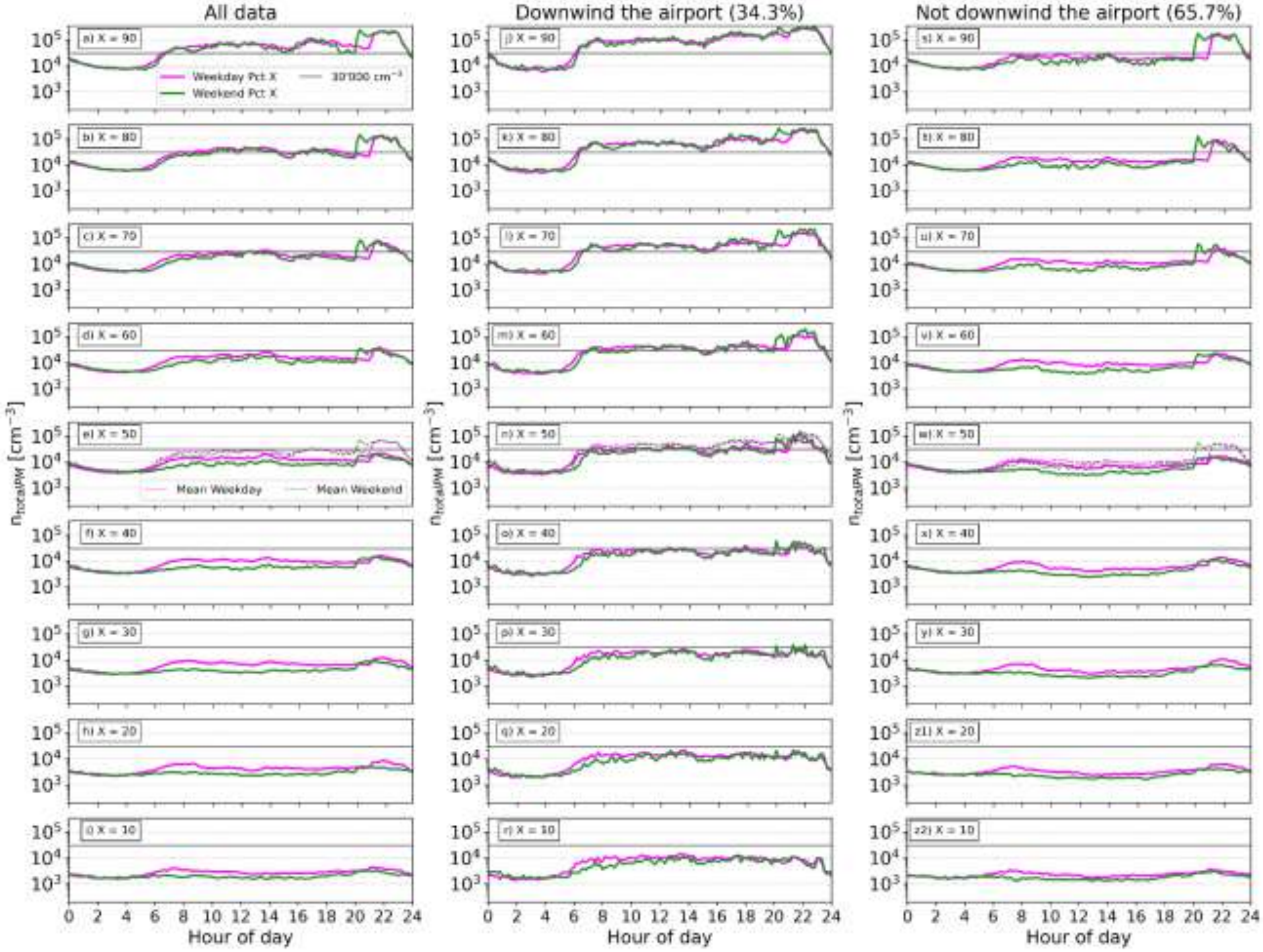

Figure S9: from top to bottom: Daily cycle of UFP's number concentration from the long term (2022 to 2023 included) dataset for different decreasing percentiles, from 90th to 10th, for all data, downwind and not downwind data. For each panel, weekdays and weekends diurnal cycles are represented. Mean values are additionally plotted as dashed lines on the panels for the 50th percentile. A horizontal grey line represents on every panel a UFPs number concentration of 30 000 cm^{-3}, which is our criteria for high concentrations dominated by aviation emissions. Data is 10-minutes averaged. The influence of road traffic is clearly discernible in the time window from 05:00 to 10:00 a.m. as a difference between the magenta and green lines in ~70% of all data, i.e. up to the 70th percentile.

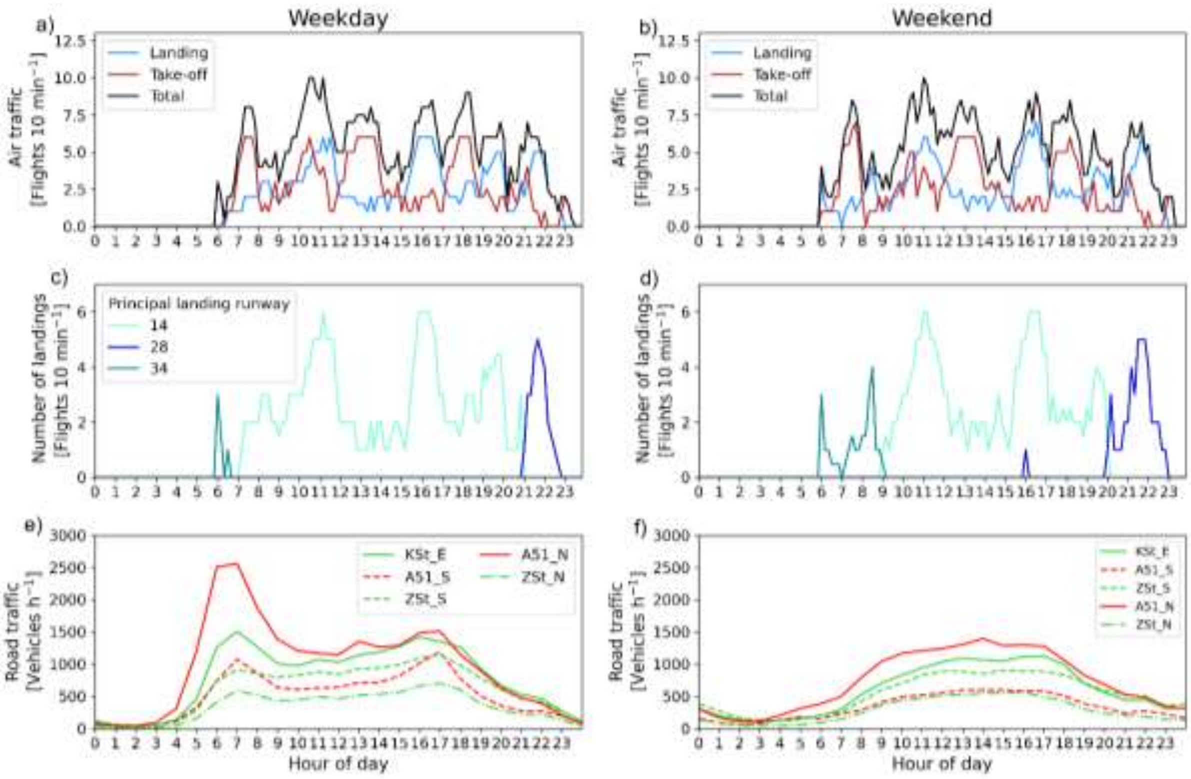

Figure S10: Left column: Diurnal cycle of a) and b) aircraft traffic, all landings and take-offs considered and c) and d) only landings for each principal landing runway shown on Fig. 1 and e) and f) hourly road traffic around the site for each measurement point described on Fig. 1, for weekdays. Right column: Same for weekends. Data is 10-minutes and 1-hour averaged for air traffic and road traffic, respectively. The air traffic consists in an alternative switch between intense take-offs and landing periods between 06:00 and shortly after 23:00. Due to noise regulations, aircraft that are usually landing on runway 14 during daytime have to land on runway 28 from 21:00 during weekdays and from 20:00 during weekends. The road traffic on major highways and national roads shows a morning rush hour between 05:00 and 09:00, only on weekdays.

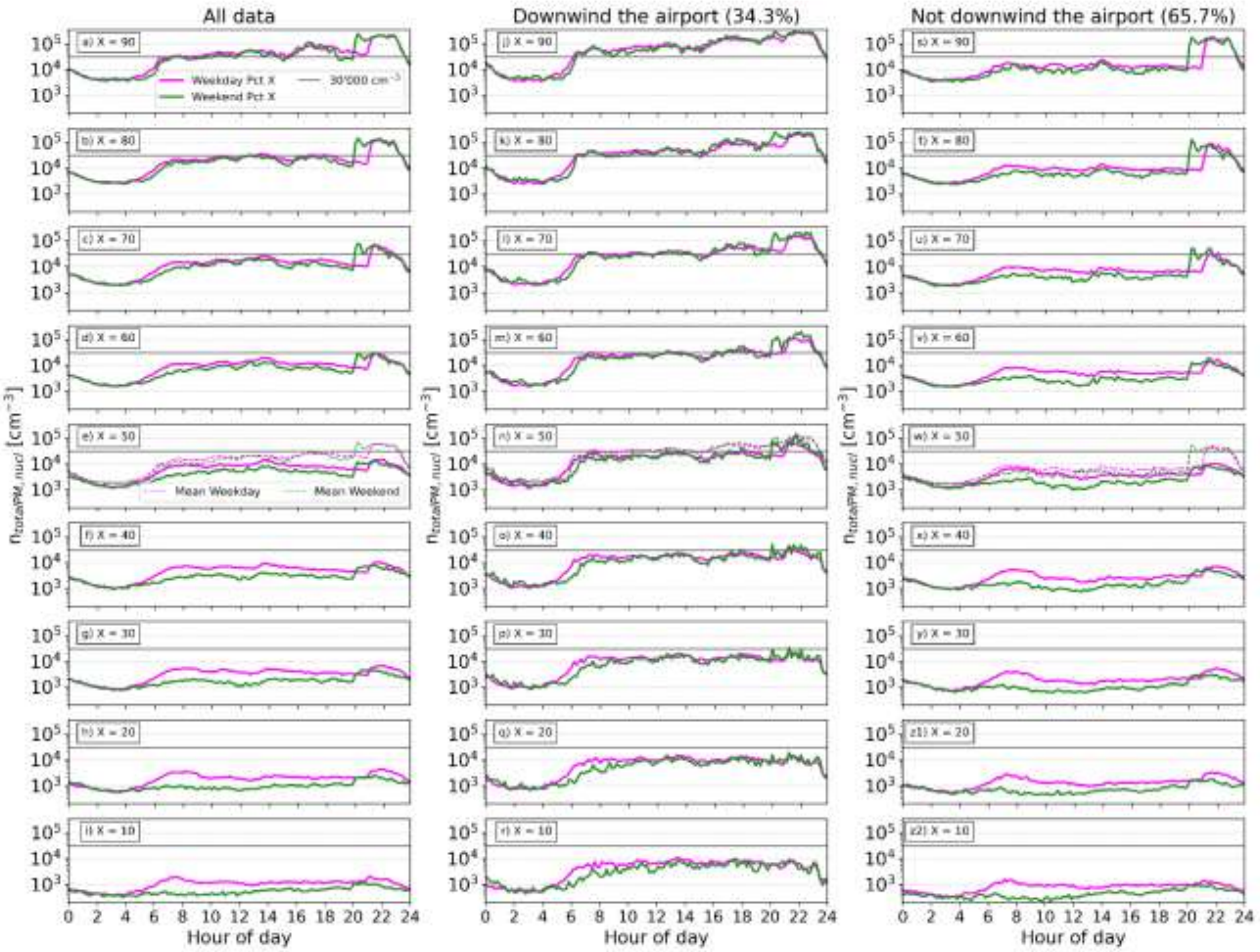

Figure S11: Same as Figure S8 but for nucleation mode UFPs, i.e. < 20 nm. A nucleation mode from road traffic is visible for at least 70 % of the data. The overall lower $n_{totalPM,nucl}$ concentrations compared to $n_{totalPM}$ during the day downwind the airport indicates a contribution of airport emissions to the Aitken mode.

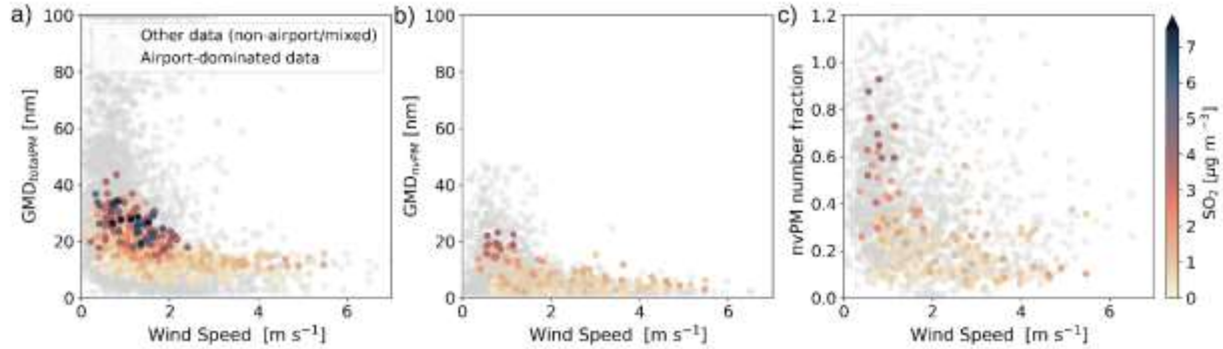

Figure S12: Same as Figure 5 in the main text, but for additionally showing data points when UFP number concentrations were < 30’000 cm^{-3} as grey data points.

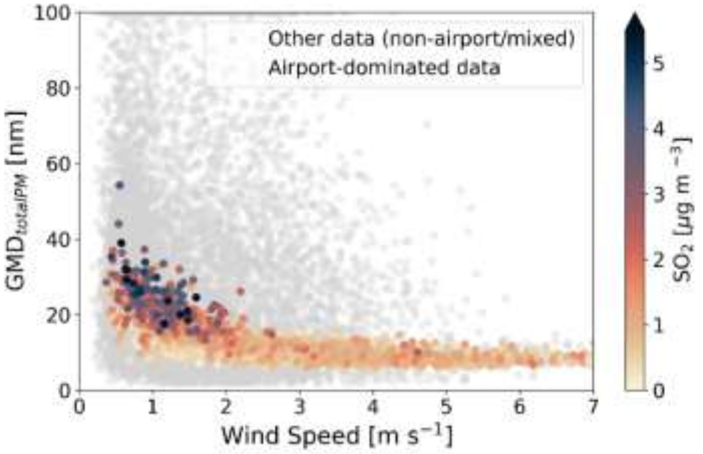

Figure S13: Same as Fig. 5a in the main text but for using the long-term dataset (2022-2023). Grey points additionally indicate data points when UFPs were not dominated by aviation emissions.

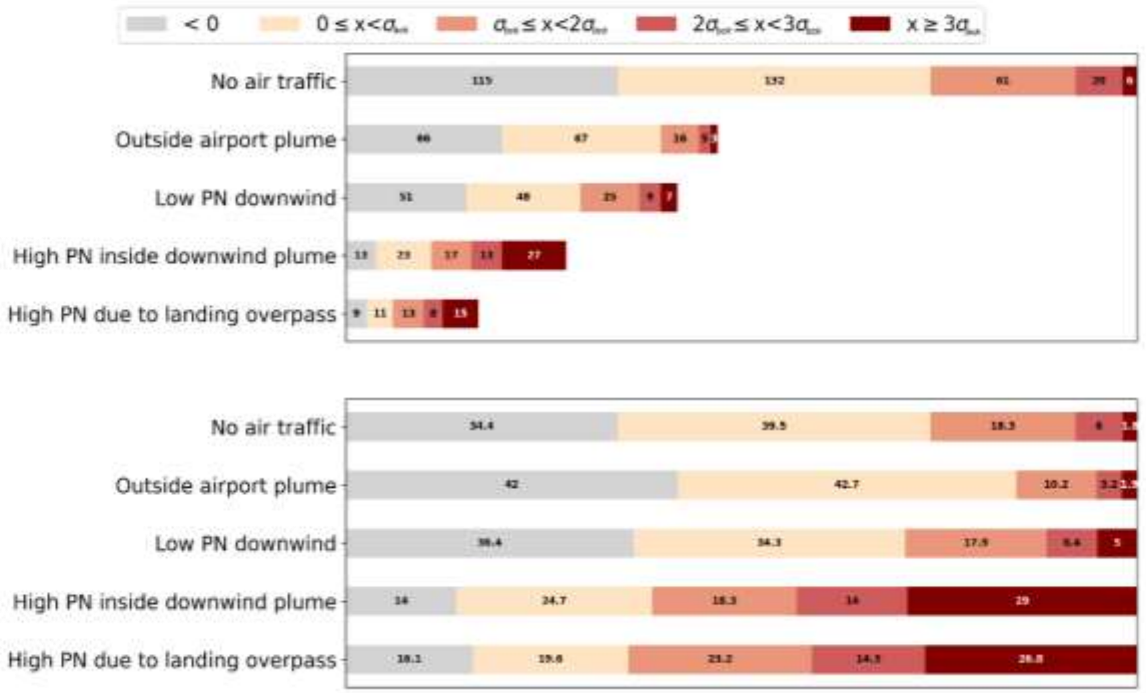

Figure S14: Occurrence of lube oil marker (TCP) signal amplitudes, which are represented as multiples of the standard deviation of the background signal. a) total count of valid data points and b) relative distribution. The 1-minute averaged dataset was further segregated by potential airport influence. The details of the criteria used to group the data into the categories on the y- axis are presented in Table S2. Numbers on the bars represent the total number of datapoint and the percentage for panels a and b, respectively. The measurements conducted during airport influence, characterized by a high PN concentration either downwind the airport or during landing overpass clearly show a higher proportion of high TCP signals. Conversely, at least 70 % of the TCP signals are not significant, i.e. negative or below σ_{bck}, when the site is not under airport influence.

	Criteria selection		
Category	Time of the day	PN concentration [cm^{-3}]	Wind speed and direction
No air traffic	00:00-06:00	/	/
Outside airport plume	06:00-19:00	/	WS > 1 m s^{-1} and WD from N to SW
Low PN downwind	06:00-19:00	< 30'000	WS > 1 m s^{-1} and WD from W to NW
High PN inside downwind plume	06:00-19:00	> 30'000	WS > 1 m s^{-1} and WD from W to NW
High PN due to landing overpass	21:00-23:00	> 30'000	/

Table S2: Criteria used to group the data by different airport influences as plotted on Fig. S14

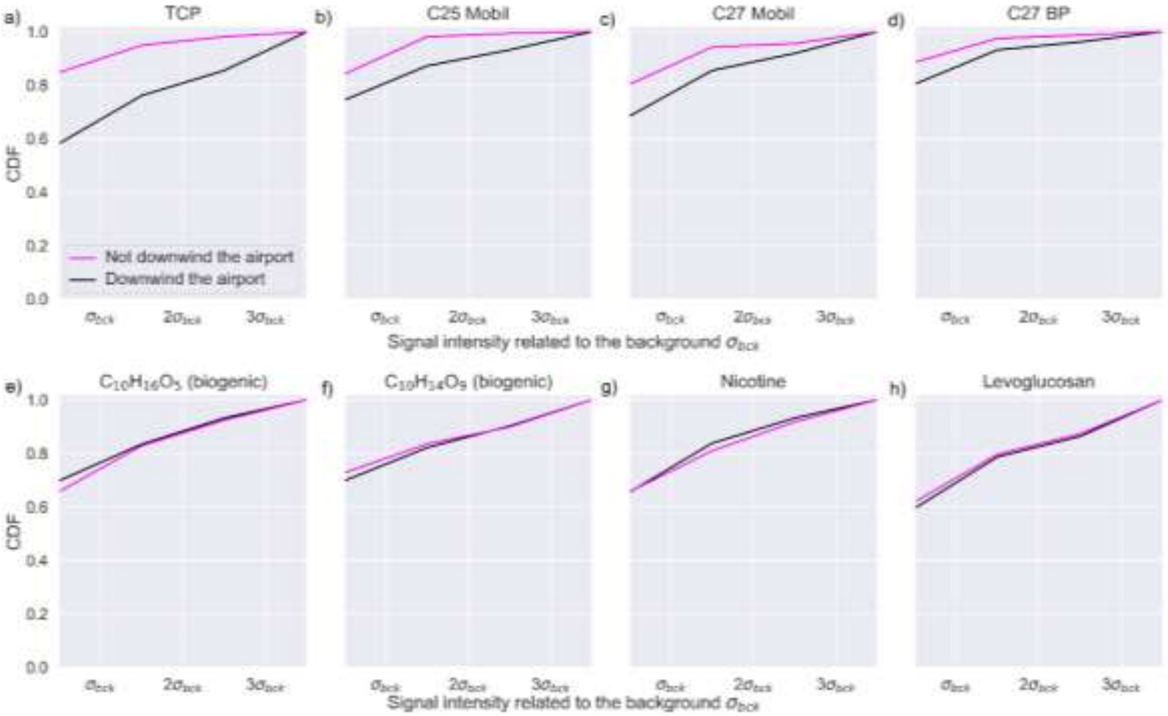

Figure S15: Downwind and not-downwind normalized cumulative distribution function (CDF) of EESI signal intensity for A) Tricresyl Phosphate, b) C25 PA Ester, c) C27 TMP Ester, d) C27 BP, e) α-Pinene hydroxy dihydroperoxide, f) α-pinene SOA g) Nicotine and h) Levoglucosan. Signal intensities have been grouped to ranges representing around one, two or three times the standard deviation (σ_{bsl}) of the background measurement of each compound. Only 1-minute averaged daytime data (from 06:00 to 19:00) with wind speeds above 1 m s^{-1} were considered to exclude stagnant conditions with poorly defined airport plume. The steeper the line, the higher the relative proportion of large signal intensities Therefore, the clear difference between the magenta and the black lines for the TCP, C25 and C27 Mobil compounds suggests that they are unique tracers of airport emissions. This interpretation is supported by absence of such a difference for the biogenic tracers (e and f). Some difference between downwind and not downwind lines were found for nicotine (cigarette smoke and traffic tracer) and levoglucosan (wood burning tracer). However, this difference is not driven by aviation emissions, as higher signals do not come with higher PN concentrations (see Figure 6 g and h).

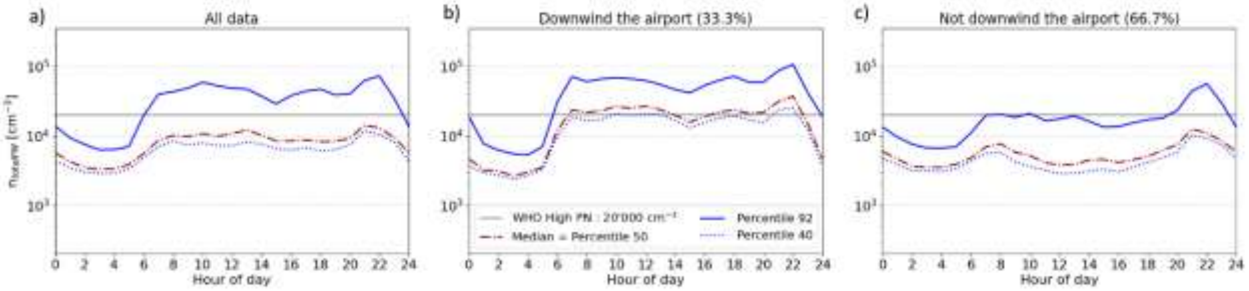

Figure S16: 1-hour averaged daily cycle of UFP's number concentration from the long term (2022 to 2023 included) dataset for percentiles 40, 50 and 92, for all data, downwind and not downwind data. The horizontal grey line represents the WHO threshold of 20'000 cm^{-3} for "high PN concentration sites", for UFPs larger than 10 nm in diameter. UFP number concentrations exceeding this threshold are to be considered as high[4]. When the site is not downwind during daytime, only 8% of the values (shown by the 92th percentile line) exceed WHO threshold Downwind the airport, which concerns one third of the measurements, 60% (shown by the 40th percentile line) of the values are above the high PN WHO threshold.

References

(1) Decker, Z. C. J.; Alpert, P. A.; Ammann, M.; Anet, J. G.; Bauer, M.; Cui, T.; Durdina, L.; Edebeli, J.; Gysel-Beer, M.; Prévôt, A. S. H.; Qi, L.; Slowik, J. G.; Spirig, C.; Tinorua, S.; Ungeheuer, F.; Vogel, A.; Zhang, J.; Brem, B. T. Emission and Formation of Aircraft Engine Oil Ultrafine Particles. *ACS EST Air* **2024**. https://doi.org/10.1021/acsestair.4c00184.

(2) Lopez-Hilfiker, F. D.; Pospisilova, V.; Huang, W.; Kalberer, M.; Mohr, C.; Stefenelli, G.; Thornton, J. A.; Baltensperger, U.; Prevot, A. S. H.; Slowik, J. G. An Extractive Electrospray Ionization Time-of-Flight Mass Spectrometer (EESI-TOF) for Online Measurement of Atmospheric Aerosol Particles. *Atmospheric Meas. Tech.* **2019**, *12* (9), 4867–4886. https://doi.org/10.5194/amt-12-4867-2019.

(3) Bell, D. M.; Zhang, J.; Top, J.; Bogler, S.; Surdu, M.; Slowik, J. G.; Prevot, A. S. H.; El Haddad, I. Sensitivity Constraints of Extractive Electrospray for a Model System and Secondary Organic Aerosol. *Anal. Chem.* **2023**, *95* (37), 13788–13795. https://doi.org/10.1021/acs.analchem.3c00441.

(4) WHO. *WHO Global Air Quality Guidelines: Particulate Matter (PM2.5 and PM10), Ozone, Nitrogen Dioxide, Sulfur Dioxide and Carbon Monoxide*; Geneva: World Health Organization, 2021.
